# Amygdalar nuclei vulnerability to protein aggregates in Lewy body diseases

**DOI:** 10.1186/s40478-026-02381-0

**Published:** 2026-07-21

**Authors:** Natasja A. C. Deshayes, Janna van Wetering, Alex Wesseling, Floris Vos, Angela Ingrassia, Yvon Galis, Laura E. Jonkman, Annemieke J. M. Rozemuller, Wilma D. J. van de Berg

**Affiliations:** 1 Section Clinical Neuroanatomy and Biobanking, Department of Anatomy and Neurosciences, Amsterdam UMC, Vrije Universiteit Amsterdam, Amsterdam, The Netherlands; 2 Amsterdam Neuroscience, Neurodegeneration Program, Amsterdam UMC, Amsterdam, The Netherlands; 3 Amsterdam Neuroscience, Brain Imaging Program, Amsterdam UMC, Amsterdam, The Netherlands; 4 Department of Pathology, Amsterdam UMC, Vrije Universiteit Amsterdam, Amsterdam, The Netherlands

**Keywords:** Alpha-synuclein, Amygdalar nuclei, Amyloid beta, Immunohistochemistry, Lewy body disease, Phosphorylated tau, Vulnerability

## Abstract

The amygdala is highly vulnerable to protein aggregation and heavily affected in Lewy body diseases (LBDs). However, vulnerability might vary per amygdalar nucleus and it is unclear if the pattern of vulnerability across the nuclei differs between types of protein aggregation and between LBDs. In this study, we aimed to assess the vulnerability of amygdalar nuclei to multiple types of protein aggregation across LBDs. Post-mortem amygdala tissue of donors with incidental LBD (iLBD, n = 6), Parkinson’s disease (PD; n = 18), dementia with Lewy bodies (DLB; n = 9) and Alzheimer’s disease with Lewy bodies (AD + LB; n = 15) was immunostained with antibodies against alpha-synuclein (aSyn; EP1536Y and 5G4), amyloid beta (Aβ; 4G8), phosphorylated tau (p-tau; AT8) and phosphorylated TDP-43 (p-TDP-43; 11–9), and quantitatively analyzed using QuPath. Neuronal and astrocytic aSyn pathology were most pronounced in the parahippocampal-amygdaloid transition area (PHA) and the basal nucleus, a pattern shared by all disease groups. Vulnerability to Aβ pathology varied per group but was highest in the PHA in AD + LB, whereas diffuse plaques were most common in the accessory basal nucleus. The PHA of DLB and both the basal and accessory basal nucleus of AD + LB cases were most susceptible to p-tau pathology, with fine granular cytoplasmic neuronal tau inclusions being mostly observed in the basal nucleus and neurofibrillary tangles in the accessory basal nucleus. The nuclei in the ventromedial part of the amygdala (PHA, ventral part of the basal nucleus, and cortical nucleus) were found to be hotspots for protein aggregation across LBDs. aSyn pathology in these nuclei predominantly correlated with dementia, hallucinations and anxiety. Our results show that amygdalar nuclei vulnerability differs per protein aggregate and disease entity, although the PHA, basal nucleus and cortical nucleus are generally more vulnerable. Together, our study provides a deeper insight into the selective vulnerability of amygdalar nuclei to protein aggregates and their relation to clinical characteristics in LBDs.

**Supplementary Information:**

The online version contains supplementary material available at https://doi.org/10.1186/s40478-026-02381-0.

## Introduction

The amygdala is an almond-shaped limbic structure, which plays an essential role in the regulation of emotions [44], associative learning [32] and social behavior [36].

The amygdala is highly susceptible to protein aggregation [39] and commonly shows Lewy body (LB) pathology in a wide variety of neurodegenerative diseases, including Parkinson’s disease (PD) [9], dementia with Lewy bodies (DLB) [46] and Alzheimer’s disease (AD) [24]. According to the brain-first model of PD, the amygdala is one of the earliest brain regions affected by LB pathology [23]. Furthermore, almost 90% of DLB cases and more than 60% of AD cases exhibit LBs in the amygdala [21, 46, 48]. Noteworthy, LB pathology in the amygdala of AD with Lewy bodies (AD + LB) donors was found to be more extensive compared to PD with dementia donors [59]. LB pathology in the amygdala in general has been associated with neuropsychiatric symptoms, including anxiety and visual hallucinations in PD [22, 30, 43, 60] and depression in AD [33].

In addition to LB pathology, the amygdala forms a preferential brain region for the aggregation of other proteins, including amyloid beta (Aβ), phosphorylated tau (p-tau) and transactive response DNA-binding protein 43 (TDP-43), in neurodegenerative diseases [39].

The amygdala consists of several nuclei, including the accessory basal, basal, central, cortical, lateral and medial nucleus [40], and the parahippocampal-amygdaloid transition area (PHA) [62]. These nuclei are highly (inter)connected; information from the primary somatosensory areas arrives at the lateral nucleus, through the thalamic medial geniculate nucleus and the dysgranular insular cortex [40]. The signals are relayed via other amygdalar nuclei to the central nucleus, which forms the output center of the amygdala [39, 63] and projects to other brain regions, including the hypothalamus and brain stem areas [39, 40], to generate an emotional response [45]. The central nucleus also projects to the locus coeruleus, which is thought to mediate stress responses [63]. The other amygdalar nuclei are also connected to other brain regions. For example, the basal nucleus is interconnected with the ventromedial prefrontal cortex and the hippocampus [6, 40, 63], and the cortical nucleus is mainly innervated by the olfactory bulb [6, 40, 53]. Whereas the connection with the hippocampus enables contextual modulation of emotional responses [53], innervation by the olfactory bulb is responsible for olfactory responses [41]. Given the widespread connections of amygdalar nuclei and their distinct functions, localization of protein aggregates within specific amygdalar nuclei may influence clinical manifestation.

Until now, only a few studies have looked into the distribution of protein aggregates across amygdalar nuclei in LBDs [15, 20, 58, 60], but these reported inconsistent findings. For example, the basal nucleus displayed most LBs in PD in one study [22], whereas the PHA and central nucleus did in two others [9, 12]. Moreover, prior research has been limited in scope, mostly examining only one or a few protein aggregates in one or two disease entities. As a result, it remains unclear whether amygdalar nuclei vulnerability patterns are protein-specific and/or nucleus-intrinsic.

To address this gap, we aimed to determine the selective vulnerability of amygdalar nuclei to multiple types of protein aggregates for four different LBDs. We included post-mortem amygdala tissue of subjects with incidental Lewy body disease (iLBD) (*n* = 6), PD (*n* = 18), DLB (*n* = 9) and AD + LB (*n* = 15) and performed immunohistochemistry for serine 129-phosphorylated aSyn (pS129-aSyn), 5G4-positive aSyn, Aβ, p-tau and phosphorylated TDP-43 (p-TDP-43) as well as Gallyas silver staining for neuritic plaques, followed by quantitative and semi-quantitative analysis using QuPath in order to map protein aggregates across nuclei and identify hotspots. To investigate the clinical relevance, we correlated pathology burden in the different nuclei with clinical characteristics and neuropsychiatric symptoms.

## Methods

### Donor inclusion and tissue processing

We included 48 brain donors with neuropathologically confirmed iLBD [10] (*n* = 6), PD (*n* = 18), DLB (*n* = 9) or AD + LB (*n* = 15), through the Netherlands Brain Bank (NBB; Amsterdam, The Netherlands (https://www.brainbank.nl/) and Normal Aging Brain Collection Amsterdam (NABCA; Amsterdam, The Netherlands (http://nabca.eu). For brain donation and the use of brain tissue as well as medical records for research purposes, a written informed consent was signed by the donors or their next of kin. The procedures for autopsy and tissue collection of the NBB and NABCA were approved by the Medical Ethical Committee of Amsterdam UMC (formerly known as VUmc; NBB 2009.148; NABCA 2018.150).

Neuropathological diagnosis of the donors had been assessed by an experienced neuropathologist (A.R.) according to the international guidelines of the Brain Net Europe consortium (https://www.brainbank.nl/about-us/brain-net-europe/) [2–4], the NIA-AA criteria for AD [37] and the DLB consortium guidelines for DLB [35]. Presence of age-related tau astrogliopathy (ARTAG) was based on the criteria described in [26].

Demographic and clinical characteristics, including sex, age at death, disease duration as well as the presence or absence of parkinsonism, dementia and neuropsychiatric symptoms, were extracted from the medical records of the donors. Clinical Dementia Rating (CDR) scores were derived from the Clinical Diagnostic Protocol. Definitions of clinical characteristics and neuropsychiatric symptoms are provided in Supplementary Table 1. Information on neuropsychiatric symptoms was collected retrospectively and when it was not available, it was recorded as ‘no’.

Tissue from the amygdala of the right hemisphere was obtained at brain autopsy, fixed in 4% buffered formalin (pH 7.4) for four weeks and embedded in paraffin. Formalin-fixed and paraffin-embedded (FFPE) amygdala tissue was then cut into 6 micron-thick sections using a paraffin microtome (Leica RM2245, Wetzlar, Germany) and mounted onto SuperFrost™ Plus adhesion microscope slides (Thermo Fisher Scientific, Massachusetts, USA).

### Histology and immunohistochemistry

For all stainings, FFPE sections were first deparaffinized by heating at 58°C, and immersion in xylene substitute (Sigma-Aldrich, Cat. A5597) and a series of decreasing concentrations of alcohol.

Nissl staining was applied to enable neuroanatomical delineation of amygdalar nuclei. For this, the sections were immersed for 30 s in thionine (Acros Ogranics, Cat. 420880250), dipped in demi water and immersed in 70%, 80% and 96% ethanol, for 1 min each. To differentiate, sections were dipped 10 times in 96% ethanol supplied with a few drops of acetic acid (Sigma-Aldrich, Cat. A6283). For dehydration and subsequent clearing, sections were immersed in 96% ethanol, two changes of 100% ethanol and three changes of xylene (Boom, Cat. 76051819.1000), before coverslipping.

The Gallyas silver staining was based on [18], which has been described before [19], and was applied to 31 of the 48 cases to visualize neuritic plaques [57].

For immunostainings for aSyn (pS129; EP1536Y, Abcam, 1:8000 dilution and aggregated aSyn; 5G4, Millipore), Aβ (4G8, BioLegend, 1:4000 dilution), p-tau (AT8, ThermoScientific, 1:800 dilution) and p-TDP-43 (11-9, Cosmo Bio, 1:4000 dilution), the procedure was described previously [59]. Specifications of the procedure and used antibodies are listed in Supplementary Table 2. EP1536Y was used to visualize (phosphorylated) neuronal aSyn [13], whereas 5G4 was used to reveal astrocytic aSyn [27, 54] in addition to neuronal inclusions.

For the Gallyas silver staining and all immunostainings, sections were counterstained with Mayer’s hematoxylin (Baker, Cat. 3870) for 90 s. Lastly, they were dehydrated with increasing concentrations of alcohol, cleared in xylene and coverslipped using Entellan (Sigma-Aldrich, Cat. 1.07960).

### Morphological and quantitative analysis and hotspot determination

Nissl-, Gallyas- and immunostained sections were scanned by a whole slide scanner (Olympus VS200) using the UPLXAPO 20x/0.80 objective. In these scans, the amygdala was manually segmented (by N.D. with help from A.W.) into the accessory basal, basal, central, cortical, lateral and medial nucleus and the parahippocampal-amygdaloid transition area in QuPath (version 0.5.0) [5] according to the Atlas of the Human Brain [34], using either the Nissl-stained section or the hematoxylin channel as a structural reference (Supplementary Fig. 1). The medial nucleus and PHA were not present in all tissue sections due to anatomical differences in sectioning level between donors. Availability of amygdalar nuclei annotations per section and donor is listed in Supplementary Table 3.

The burden of pSer129-aSyn, 5G4-positive aSyn, p-tau, Aβ and p-TDP-43 was quantified as % area of DAB-positive immunoreactive signal in QuPath, by applying in-house developed scripts that make use of pixel classifiers, which were optimized for each immunostaining (Supplementary Fig. 2).

For both semi-quantitative morphological analysis and hotspot determination, the entire amygdala was overlaid by a grid of tiles of 250 000 µm^2^ (for aSyn, p-tau and p-TDP-43) or 640 000 µm^2^ (for Aβ and Gallyas) in QuPath (Supplementary Fig. 3). In the Gallyas-stained sections, multiple tiles of 640,000 µm^2^ per nucleus were placed randomly (Supplementary Table 4). The tile displaying the highest immunoreactivity within each nucleus was subjected to morphological analysis, whereas the nucleus in which the tile with the highest immunoreactivity was located was assigned the hotspot for that donor for that type of pathology. For the basal and lateral nucleus, a distinction was made between ventral, middle and dorsal.

Definitions of all morphological structures and quantification units are given in Supplementary Table 5. Ordinal scoring of astrocytic aSyn morphologies (Otero-Jimenez, Wojewska [42]) is explained in Supplementary Fig. 4. The semi-quantitative morphological analysis was conducted by N.D. with J.W. as a second assessor, who were both blinded to the neuropathological diagnoses, and yielded an Intraclass Correlation Coefficient of 1 for p-TDP-43 neuronal cytoplasmic inclusions (NCIs), 0.97 for diffuse plaques and fine granular cytoplasmic neuronal tau inclusions, 0.96 for LBs and neurofibrillary tangles and 0.82 for cored plaques.

### Statistical analysis

All statistical tests were performed in RStudio (version 4.3.2). Differences in categorical variables between groups were calculated using the Fisher’s exact test, followed by the pairwise Fisher’s test with Bonferroni correction. For differences in continuous variables, ANOVA with Tukey post-hoc test was used. When assumptions for normality and/or homogeneity of variance were not met (as tested by the Shapiro–Wilk and Levene’s test, respectively), as well as for CDR and pathological stages, group differences were evaluated using the Kruskal–Wallis test with subsequent Wilcoxon rank-sum tests and Benjamini–Hochberg or Bonferroni correction for multiple comparisons. To assess differences in burden or density of protein aggregates (in total % area and number per 0.25 mm^2^, respectively) between nuclei, a linear mixed model was applied including nucleus, group, their interaction, batch (when applicable), gender and age at death as fixed effects and with a random intercept per donor to account for nestedness of data. Burden of pS129-aSyn, Aβ and p-tau were log-transformed (for p-tau 0.01 was added as a constant) to improve normality, whereas a squared root transformation was applied to 5G4-positive aSyn burden. For count data, a general linear mixed model with negative binomial family (type 1 for diffuse plaques, and type 2 for LBs, cored plaques, fine granular cytoplasmic neuronal tau inclusions, and neurofibrillary tangles) was used. Due to convergence issues with the full model for the density of p-TDP-43 NCIs and processes, which likely reflect the substantial proportion of zero values, the Kruskal–Wallis test was used instead. Consequently, covariate adjustment was not possible, but the influence of confounders on between-nuclei comparisons was assumed to be limited as their covariate distribution was inherently balanced across nuclei. Differences in ordinal scores between nuclei were modelled using a cumulative logit mixed model but this was only possible for the DLB and AD + LB groups. Correlations between pathological (pS129-aSyn and 5G4-positive aSyn pathology) and clinical (disease duration, dementia, CDR, hallucinations, anxiety and depression) characteristics across all groups were assessed by pairwise Spearman correlation analyses, and corresponding p-values were corrected for multiple comparisons using the False Discovery Rate (FDR). Graphs were also generated in RStudio but edited and/or merged in Adobe Illustrator 2025 (Adobe Inc., San José, California, USA).

## Results

### Clinical and pathological characteristics of brain donors

The demographic, clinical and neuropathological characteristics of all included donors are summarized in Table 1. For data on individual donors, see Supplementary Table 6 and Supplementary Table 7. In summary, iLBD donors were older at death (82 ± 6.7 years) compared to DLB donors (70 ± 6.2 years, *p* = 0.015) and AD + LB donors (73 ± 7.6 years, *p* = 0.049). PD donors had a longer disease duration (15 ± 7.8 years) than DLB (7 ± 3.3 years, *p* = 0.007) and AD + LB (8 ± 4.5 years, *p* = 0.006) donors. Parkinsonism was present in the PD (100%), DLB (89%) and AD + LB (60%) groups. Thirty-six out of forty-eight donors (75%) showed dementia, including all DLB and AD + LB donors and more than half (61%) of the PD donors. Per definition, iLBD cases did not show parkinsonism or dementia [1]. Consequently, dementia prevalence was significantly higher among DLB and AD + LB cases (*p* = 0.001 and *p* < 0.05, respectively) and parkinsonism was significantly more prevalent among PD and DLB donors (*p* < 0.001 and *p* = 0.018, respectively) compared to iLBD donors. In contrast to dementia prevalence, there were no significant differences in CDR scores between groups. Hallucinations were experienced more frequently by PD (89%), DLB (100%) and AD + LB (73%) cases than by iLBD cases (*p* = 0.004, *p* = 0.003 and *p* = 0.049, respectively). Anxiety and depression were not significantly different among groups (*p* = 0.132 and *p* = 0.071, respectively). Braak LB stages of PD, DLB and AD + LB donors were higher compared to iLBD donors (*p* < 0.001, *p* = 0.032 and *p* = 0.004, respectively). As expected, AD + LB donors had a higher Braak NFT stage and Thal phase compared to all other groups (all *p* < 0.047). There were no differences in CAA type (*p* = 0.109) and the prevalence of ARTAG among groups (*p* = 0.16 to *p* = 1.00).

**Table 1:** Clinical and pathological characteristics of included brain donors

	iLBD	PD	DLB	AD + LB
*Demographic characteristics*				
N	6	18	9	15
Sex F/M (%F)	3/3 (50%)	7/11 (39%)	1/8 (11%)	6/9 (40%)
Age at death in years, mean ± SD [range]	82 ± 6.7 [72–89]	77 ± 7.8 [60–89]	70 ± 6.2 [58–79]*	73 ± 7.6 [56–81]*
*Clinical characteristics*				
Age at disease onset in years, mean ± SD [range]	NA	62 ± 11.0 [41–81]	63 ± 7.1 [52–74]	65 ± 10.1 [49–79]
Disease duration in years, mean ± SD [range]	NA	15 ± 7.8 [6–32]	7 ± 3.3 [4–15]^#^	8 ± 4.5 [1–16]^#^
PMD in hours:min, mean [range]	6:50 [4:45–8:25]	5:45 [3:35–9:17]	6:00 [4:25–8:15]	5:50 [3:35–9:50]
Parkinsonism yes/no (%yes)	0/6 (0%)	18/0 (100%)*	8/1 (89%)*	9/6 (60%)^#^
Dementia yes/no (%yes)	0/6 (0%)	11/7 (61%)	9/0 (100%)*	15/0 (100%)*
CDR 0/0.5/1/2/3/unknown	1/0/0/0/0/5	1/4/2/3/5/3	0/1/1/2/2/3	0/0/0/2/5/8
*Neuropsychiatric characteristics*				
Hallucinations yes/not mentioned (%yes)	0/6 (0%)	16/2 (89%)*	9/0 (100%)*	11/4 (73%)*
Anxiety yes/not mentioned (%yes)	1/5 (17%)	7/11 (39%)	7/2 (78%)	9/6 (60%)
Depression yes/not mentioned (%yes)	2/4 (33%)	7/11 (39%)	8/1 (89%)	8/7 (53%)
*Neuropathological characteristics*				
Braak LB stage 0/1/2/3/4/5/6/atypical	0/0/0/0/4/1/1	0/0/0/0/0/3/15/0*	0/0/0/0/1/2/6/0*	0/0/0/0/1/1/11/2*
Braak NFT stage 0/1/2/3/4/5/6	1/3/1/1/0/0/0^$^	1/5/8/4/0/0/0^$^	0/3/2/4/0/0/0^$^	0/0/0/0/2/8/5
Thal phase 0/1/2/3/4/5/unknown	1/4/1/0/0/0/0^$^	4/6/1/7/0/0/0^$^	0/1/2/3/2/0/1^$^	0/0/0/3/9/2/1
CAA type absent/1/2/unknown	6/0/0/0	12/1/5/0^$^	4/2/3/0	7/2/3/3
ARTAG yes/no (%yes)	0/6 (0%)	8/10 (44%)	4/5 (44%)	5/10 (33%)
LATE-NC stage	0/1/0/0/5	11/0/0/0/7	1/1/0/0/7	4/2/0/0/9
0/1/2/3/unknown (%yes)	(17%)	(0%)	(11%)	(13%)

*Significantly different from iLBD

^#^Significantly different from PD

^$^Significantly different from AD + LB

AD+LB: Alzheimer’s disease with Lewy bodies; ARTAG: age-related tau astrogliopathy; CAA: cerebral amyloid angiopathy; DLB: dementia with Lewy bodies; F: female; iLBD: incidental Lewy body disease; LB: LATE-NC: limbic-predominant age-related TDP-43 encephalopathy neuropathological change; Lewy body; M: male; NA: not applicable; NFT: neurofibrillary tangle; PD: Parkinson’s disease; PMD: post-mortem delay

### PHA and basal nucleus are most vulnerable to aSyn pathology

**Fig. 1:**
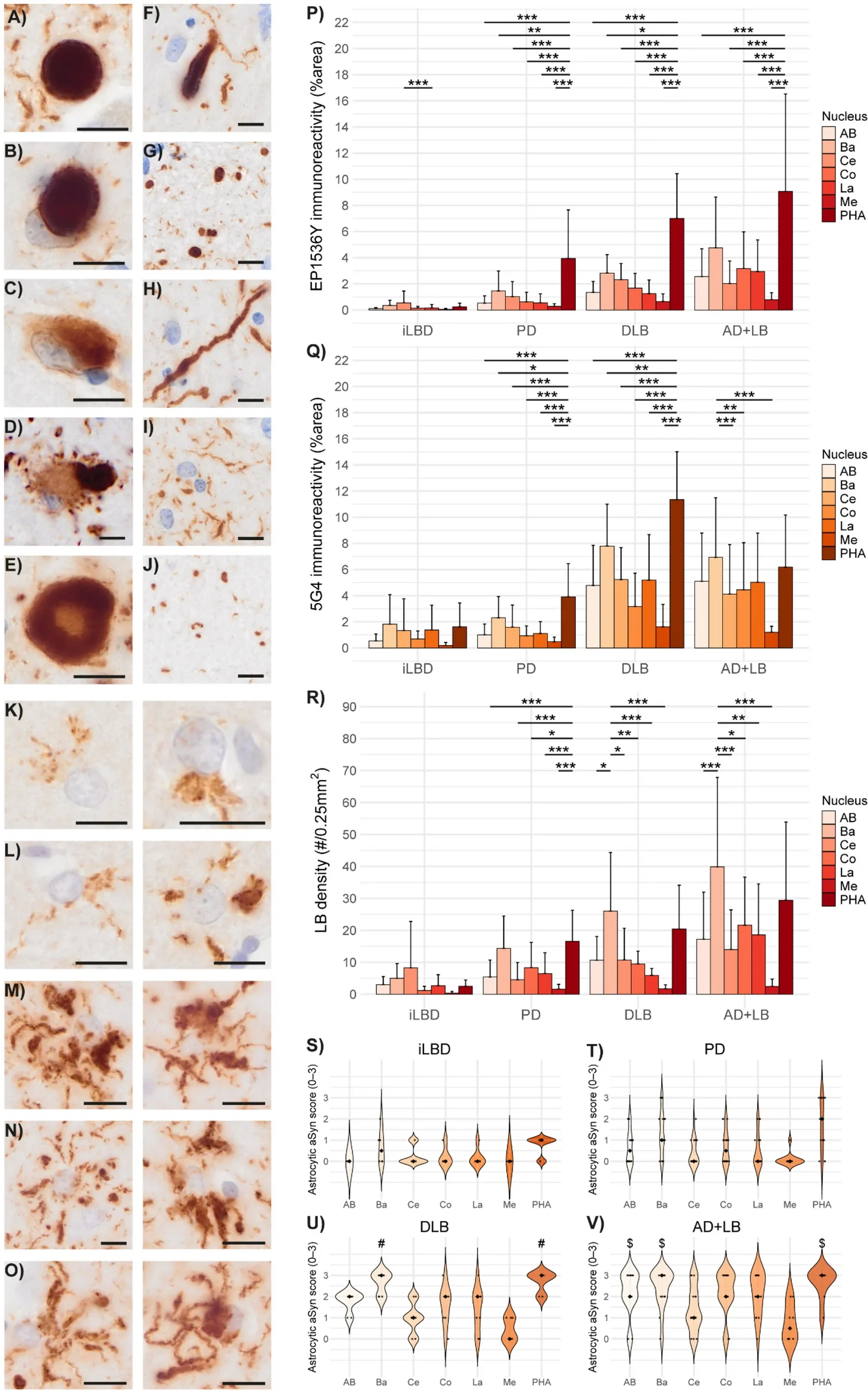
The PHA and basal nucleus are selectively vulnerable to aSyn pathology. **A**–**J** Morphological structures of pS129-aSyn pathology observed include extracellular LBs (**A**), intracellular LBs (**B**), diffuse cytoplasmic LBs (**C**), granular inclusions (**D**), axonal swellings (**E**), bulgy and Lewy neurites (**F**–**H**), Lewy threads (**I**) and synaptic staining (**J**). **K**–**O** Examples of 5G4-positive aSyn morphologies, including grainy (**K**), wispy (**L**), spider (**M**), star-like (**N**) and spiky (**O**) astrocytic inclusions, the latter three of which are quantified in S-V. Scale bar represents 10 µm. **P**–**R** Quantification of pS129-aSyn pathology (total % area of EP1536Y immunoreactivity) (**P**), 5G4-positive aSyn pathology (total % area of 5G4 immunoreactivity) (**Q**) and LB density (in number per 0.25 mm^2^) (**R**) per amygdalar nucleus for iLBD, PD, DLB and AD + LB. Data are shown as mean ± SD. Only statistically significant differences involving the nucleus with the highest immunoreactivity within each group are shown. P-values for all pairwise comparisons are tabulated in Supplementary Table 8. *means *p* < 0.05, **means *p* < 0.01 and *** means *p* < 0.001. **S**–**V** Violin plots displaying the distribution of astrocytic aSyn score for each nucleus for iLBD (**S**), PD (**T**), DLB (**U**) and AD + LB (**V**) with the black diamond representing the median. # means *p* < 0.05 compared to Ce, Co, La and Me and $ means *p* < 0.05 compared to Ce and Me. AB: accessory basal nucleus; AD + LB: Alzheimer’s disease with Lewy bodies; Ba: basal nucleus; Ce: central nucleus; Co: cortical nucleus; DLB: dementia with Lewy bodies; iLBD: incidental Lewy body disease; La: lateral nucleus; LB: Lewy body; Me: medial nucleus; PD: Parkinson’s disease; PHA: parahippocampal-amygdaloid transition area

In order to assess the vulnerability of amygdalar nuclei to aSyn pathology, we evaluated the morphological structures as well as the burden of both pS129-aSyn and 5G4-positive aSyn pathology across amygdalar nuclei. A wide variety of pS129-aSyn-positive morphological structures were detected, including LBs (Fig. 1A–D; quantification in Fig. 1L), axonal swellings (Fig. 1E), bulgy and Lewy neurites (Fig. 1F–H), Lewy threads (Fig. 1I) and synaptic staining (Fig. 1J). Moreover, the central nucleus often exhibited granular cytoplasmic inclusions (Fig. 1D). The 5G4-positive aSyn staining revealed, in addition to neuronal inclusions, diverse astrocytic inclusions (Fig. 1K–O), including spider, star-like and spiky structures.

When all pS129-aSyn-positive morphological structures were taken together (total %area), the central nucleus of iLBD cases (*p* < 0.001 vs. lateral nucleus) and the PHA of PD, DLB and AD + LB cases (*p* = 0.019 to *p* < 0.001) were found to be the most vulnerable (Fig. 1P). P-values for all pairwise comparisons are listed in Supplementary Table 8. In all symptomatic groups, the basal nucleus and the PHA were the most vulnerable to 5G4-positive aSyn pathology (*p* = 0.030 to *p* < 0.001) (Fig. 1Q). Of note, the medial nucleus was consistently least affected by pS129-aSyn and 5G4-positive aSyn pathology in all groups.

Vulnerability to LBs varied per group (Fig. 1R): it was highest in the PHA in PD (*p* = 0.013 to *p* < 0.001) and the basal nucleus in both DLB (*p* = 0.016 to *p* < 0.001) and AD + LB (*p* = 0.013 to *p* < 0.001). Among iLBD and PD donors, the PHA showed most astrocytic aSyn inclusions, but not significantly more than other nuclei (Fig. 1S, T). In the DLB and AD + LB groups, the PHA as well as the basal nucleus were most affected by astrocytic aSyn inclusions (*p* = 0.038 to *p* < 0.001 for DLB and *p* = 0.045 to *p* = 0.002 for AD + LB) (Fig. 1U, V).

In summary, the PHA was most vulnerable to pS129-aSyn pathology, the basal nucleus to LBs, and both to 5G4-positive aSyn pathology and astrocytic aSyn inclusions. This pattern was consistent across symptomatic groups, but strongest in DLB and AD + LB.

### PHA is most vulnerable to Aβ pathology in AD + LB

**Fig. 2:**
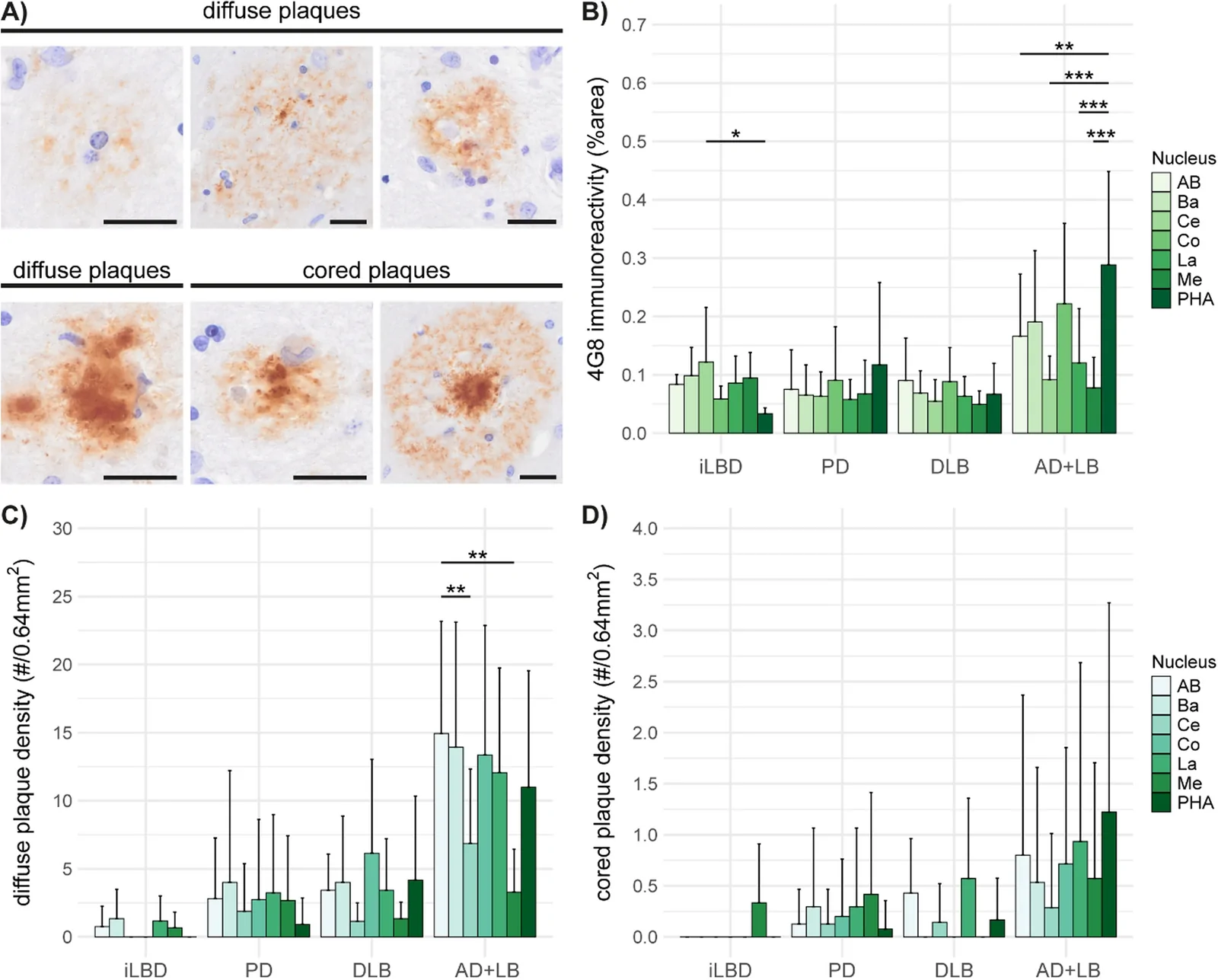
The PHA is most vulnerable to Aβ pathology and the accessory basal nucleus to diffuse plaques among AD + LB cases. **A** Examples of diffuse and cored plaques that are quantified in C and D, respectively. Scale bar represents 20 µm. **B**–**D** Quantification of Aβ pathology (total % area of 4G8 immunoreactivity) (**B**) and density of diffuse (**C**) and cored (**D**) plaques (in number per 0.64 mm^2^) per amygdalar nucleus for iLBD, PD, DLB and AD + LB. Data are shown as mean ± SD. Only statistically significant differences involving the nucleus with the highest 4G8 immunoreactivity within each group are shown. P-values for all pairwise comparisons of **B** and **C** are tabulated in Supplementary Table 9. *Means *p* < 0.05, ** means *p* < 0.01 and ***means *p* < 0.001. AB: accessory basal nucleus; AD + LB: Alzheimer’s disease with Lewy bodies; Ba: basal nucleus; Ce: central nucleus; Co: cortical nucleus; DLB: dementia with Lewy bodies; iLBD: incidental Lewy body disease; La: lateral nucleus; Me: medial nucleus; PD: Parkinson’s disease; PHA: parahippocampal-amygdaloid transition area

Two common Aβ morphological structures in the amygdala were diffuse plaques and cored plaques (Fig. 2A). Out of 45 donors, 30 (67%) showed at least one plaque type.

Whereas the central nucleus was the most vulnerable to Aβ pathology (total % area) among iLBD cases (*p* = 0.045 vs. PHA), this was the PHA among AD + LB cases (*p* = 0.008 to *p* < 0.001 vs. other nuclei) (Fig. 2B). In PD and DLB, the PHA and accessory basal nucleus, respectively, displayed the highest Aβ burden but this was not statistically significant.

Diffuse plaques were most commonly observed, namely in 50% of the iLBD, 41% of the PD, 86% of the DLB and all of the AD + LB donors. In AD + LB, the accessory basal nucleus exhibited the highest density of diffuse plaques (*p* = 0.001 vs. central nucleus and *p* = 0.002 vs. medial nucleus) (Fig. 2C).

Cored plaques were less common among iLBD (16.7%) and PD (35%) cases, but they were still present in more than half of the DLB and AD + LB cases (71% and 87%, respectively). The pattern of vulnerability to cored plaques differed from that to diffuse plaques: the medial nucleus of iLBD and PD cases, the lateral nucleus of DLB cases and the PHA of AD + LB cases showed the highest susceptibility to cored plaques (Fig. 2D). However, the differences between nuclei within groups did not reach statistical significance. In other words, amygdalar nuclei vulnerability to Aβ pathology differed between groups and was dependent on the type of plaque.

### Accessory basal and basal nucleus of AD + LB cases are most vulnerable to p-tau pathology

**Fig. 3:**
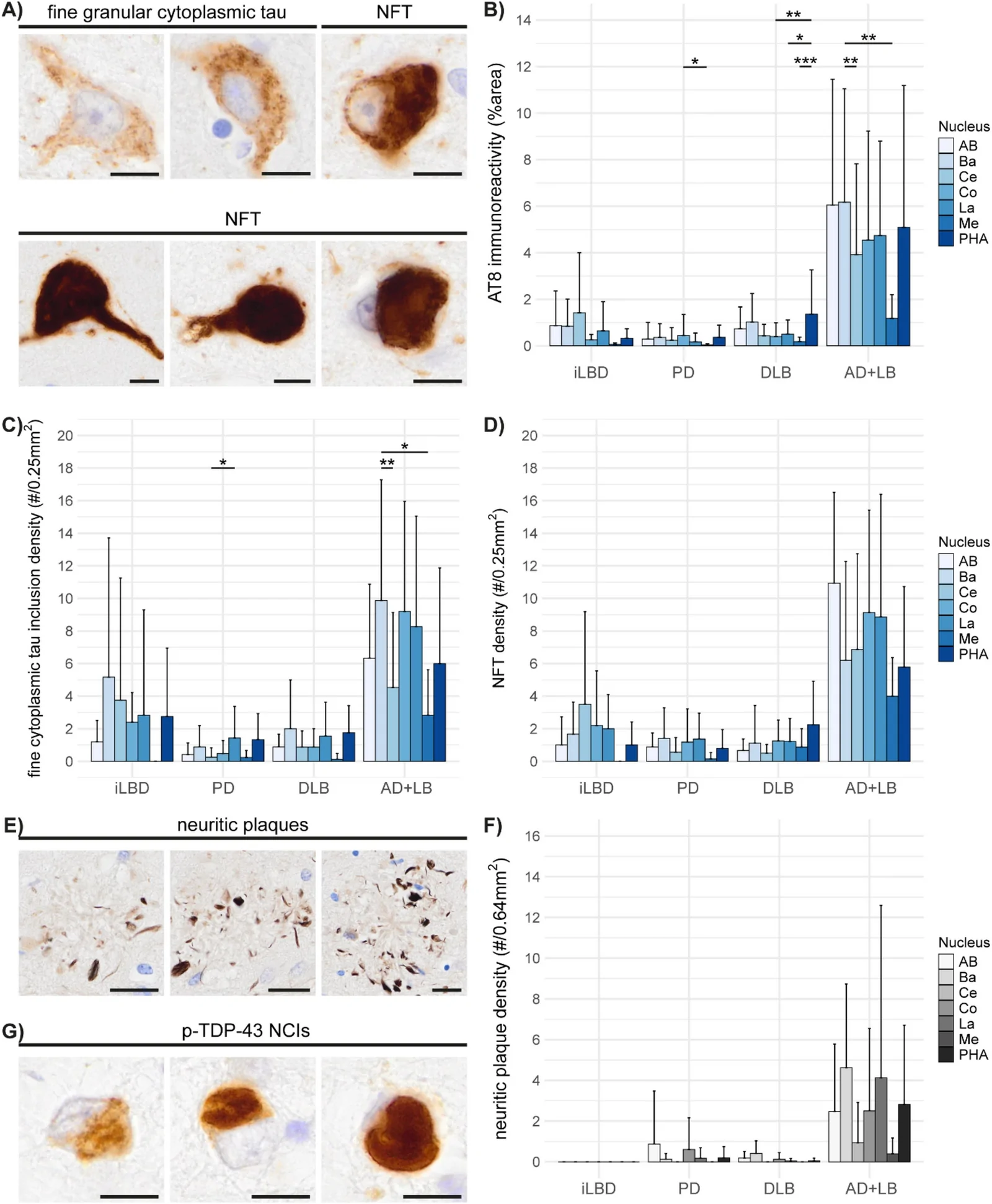
The basal nucleus of AD + LB donors is most vulnerable to overall p-tau pathology and fine granular cytoplasmic neuronal tau inclusions. **A** Examples of fine granular cytoplasmic neuronal tau and neurofibrillary tangles that are quantified in C and D, respectively. Scale bar represents 10 µm. **B**–**D**, **F** Quantification of total p-tau pathology (total % area of AT8 immunoreactivity) (**B**) and density of fine granular cytoplasmic neuronal tau inclusions (**C**) and neurofibrillary tangles (**D**) (in number per 0.25 mm^2^) and neuritic plaques (in number per 0.64 mm^2^) (**F**) per amygdalar nucleus for iLBD, PD, DLB and AD + LB. Data are shown as mean ± SD. Only statistically significant differences involving the nucleus with the highest AT8 immunoreactivity within each group are shown. P-values for all pairwise comparisons of B and C are tabulated in Supplementary Table 9.*Means *p* < 0.05, ** means *p* < 0.01 and ***means *p* < 0.001. **E** Examples of neuritic plaques that are quantified in F. Scale bar represents 20 µm. AB: accessory basal nucleus; AD + LB: Alzheimer’s disease with Lewy bodies; Ba: basal nucleus; Ce: central nucleus; Co: cortical nucleus; DLB: dementia with Lewy bodies; iLBD: incidental Lewy body disease; La: lateral nucleus; Me: medial nucleus; NCI: neuronal cytoplasmic inclusion; NFT: neurofibrillary tangle; PD: Parkinson’s disease; PHA: parahippocampal-amygdaloid transition area

Different types of p-tau inclusions were observed in the amygdala of all groups but the most prominent were fine granular cytoplasmic neuronal tau inclusions and neurofibrillary tangles (Fig. 3A).

When it comes to p-tau pathology (total % area), the cortical nucleus of PD donors (*p* = 0.034 vs. medial nucleus), PHA of DLB donors (*p* = 0.024 to *p* < 0.001), and basal nucleus of AD + LB donors (*p* = 0.007 to *p* = 0.002) were found to be most vulnerable (Fig. 3B).

Fine granular cytoplasmic neuronal tau inclusions were observed in 87% and neurofibrillary tangles in 96% of all donors. The lateral nucleus of PD donors (*p* = 0.038 vs. central nucleus) and the basal nucleus of AD + LB donors (*p* = 0.001 versus central nucleus and *p* = 0.038 vs. medial nucleus) were the most susceptible to fine granular cytoplasmic neuronal tau inclusions (Fig. 3C). There were no statistically significant differences in the density of neurofibrillary tangles between nuclei within groups (Fig. 3D).

Neuritic plaques (Fig. 3E) were found in 44% of the PD, 43% of the DLB and all of the AD + LB cases but not in iLBD cases. However, neuritic plaque density did not significantly differ across nuclei either (Fig. 3F).

### There are no differences in amygdalar nucleus vulnerability to p-TDP-43 inclusions and processes

Regarding p-TDP-43 pathology, well over one-third of the donors displayed more than one neuronal cytoplasmic inclusion (NCI) (Fig. 3G), corresponding to 1/6 iLBD, 2/9 PD, 3/9 DLB and 7/10 AD + LB donors. The highest NCI density was found in the PHA among DLB cases, and in the basal nucleus among AD + LB cases, but this was not statistically significantly different from other nuclei within the same groups. In addition to NCIs, p-TDP-43 processes had been quantified. Again, more than one-third of the cases showed at least one p-TDP-43 process, namely 1/6 iLBD, 1/9 PD, 3/7 DLB and 7/10 AD + LB cases. The density of p-TDP-43 processes was highest in the accessory basal nucleus of AD + LB cases, but this did not reach statistical significance either (data not shown).

### The ventromedial amygdala is a hotspot for protein aggregates

**Fig. 4:**
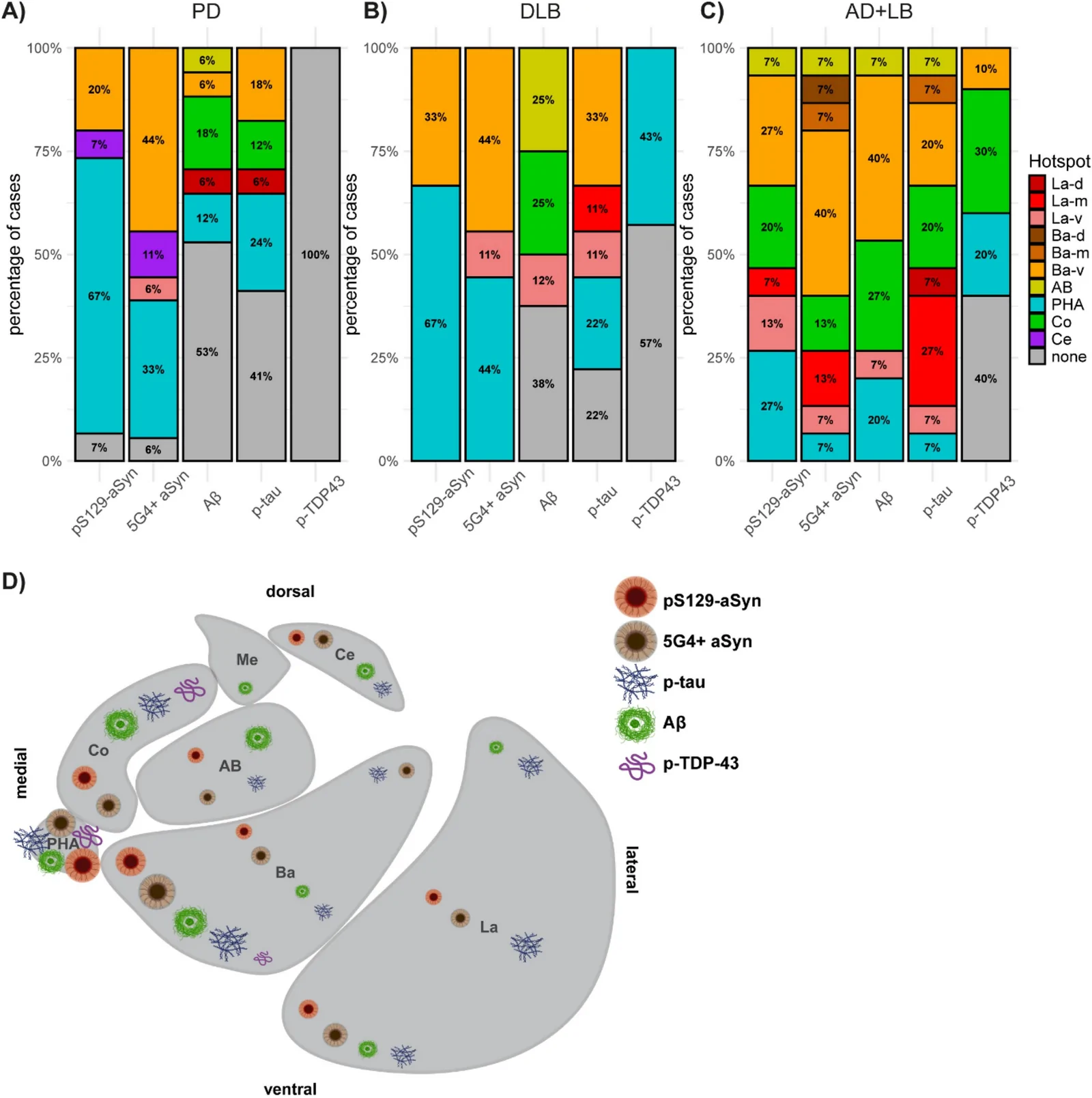
The PHA, ventral basal nucleus and cortical nucleus form hotspots for protein pathology. **A**–**D** Stacked bar graphs show which amygdalar nuclei are hotspots for pS129-aSyn, 5G4-positive aSyn, Aβ, p-tau and p-TDP-43 pathology and for which percentage of PD donors (**A**), DLB donors (**B**) or AD + LB donors (**C**). **D** shows a graphical summary of A–C, in which the size of the symbols reflects the frequency of that nucleus being a hotspot for that specific type of pathology. Aβ: amyloid beta; AB: accessory basal nucleus; AD + LB: Alzheimer’s disease with Lewy bodies; aSyn: alpha-synuclein; Ba: basal nucleus; Ce: central nucleus; Co: cortical nucleus; DLB: dementia with Lewy bodies; La: lateral nucleus; Me: medial nucleus; PD: Parkinson’s disease; PHA: parahippocampal-amygdaloid transition area; pS129: phosphorylated serine 129; p-tau: phosphorylated tau; p-TDP-43: phosphorylated transactive response DNA-binding protein-43; −d: dorsal part; m: middle part; −v: ventral part

We then identified for each case per type of protein pathology in which nucleus (part) the highest burden was observed, referred to as “hotspot”. The pattern of hotspots differed per disease group. In the PD group, the PHA was the hotspot for pS129-aSyn pathology in two-thirds (67%) and for p-tau pathology in almost a quarter (24%) of the donors (Fig. 4A). 5G4-positive aSyn and Aβ pathology hotspots were most frequently located in the ventral part of the basal nucleus (44%) and cortical nucleus (18%), respectively. Only for some PD donors, the central nucleus was identified as a hotspot for both types of aSyn pathology. In DLB, the PHA also emerged as the pS129-αSyn pathology hotspot for two-thirds (67%) of the cases (Fig. 4B). Both the PHA and ventral basal nucleus were common hotspots for 5G4-positive aSyn pathology (44%) and the latter was also considered the p-tau pathology hotspot in one-third (33%) of the DLB cases. When it comes to Aβ pathology, the accessory basal and cortical nucleus often emerged as hotspots, together accounting for half of the cases (25% each). The dorsal part of the lateral nucleus was, however, most frequently identified as the p-TDP-43 pathology hotspot (22%). Among AD + LB donors, the ventral part of the basal nucleus as well as the cortical nucleus were designated as most prevalent hotspots for all types of pathology except p-tau, for which the medial part of the lateral nucleus was found to be the most common hotspot (Fig. 4C). Taken together, the ventromedial side of the amygdala, consisting of the PHA, ventral part of the basal nucleus and the cortical nucleus, was selectively vulnerable to all types of protein pathology in most cases (Fig. 4D).

### aSyn pathology in hotspot nuclei correlated with dementia, hallucinations and anxiety

**Fig. 5:**
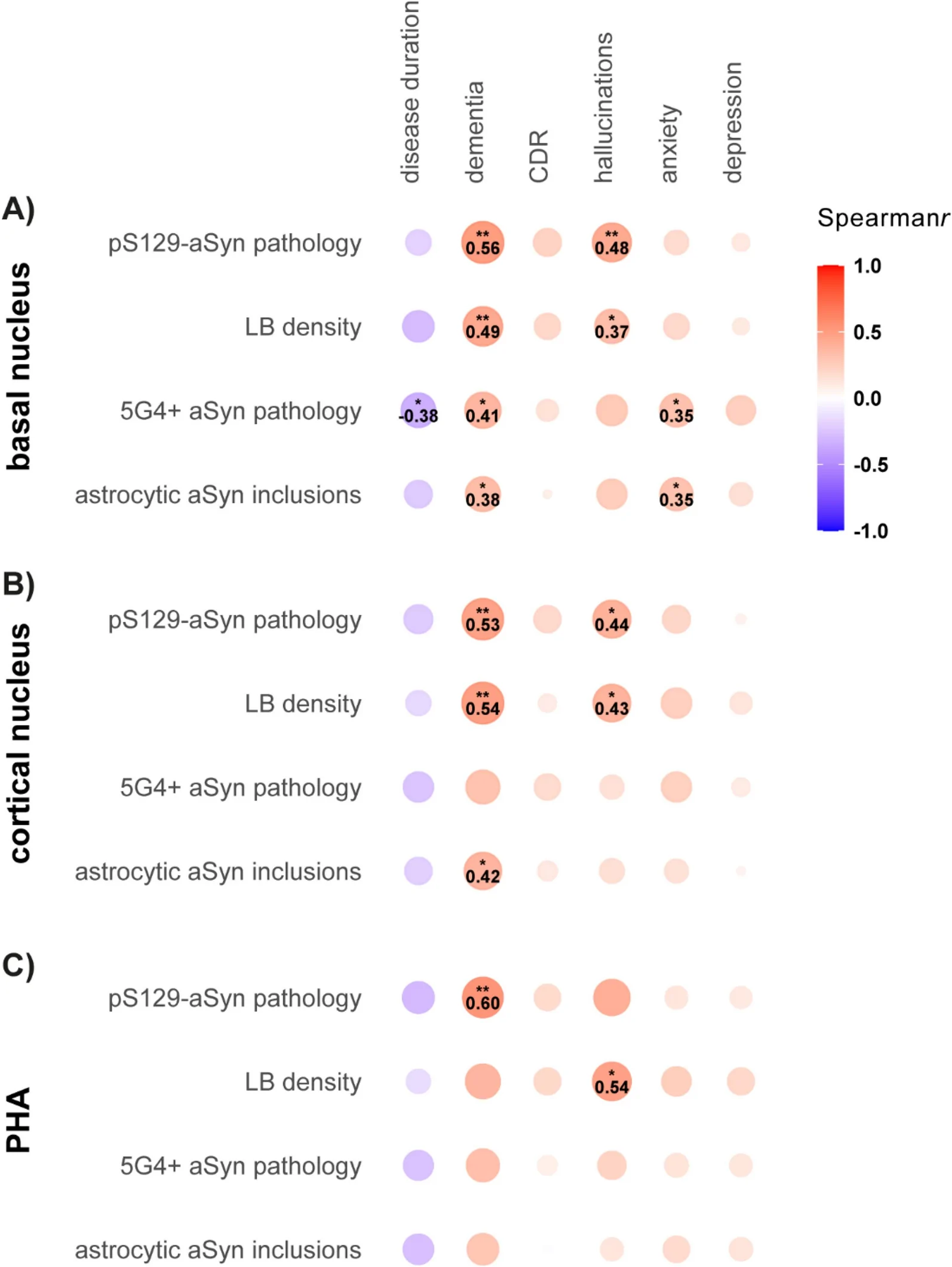
aSyn pathology in the amygdala hotspot nuclei correlates with clinical characteristics and neuropsychiatric symptoms. **A**–**C** Bubble plots show pairwise Spearman correlations between clinical characteristics and pathological features of the basal nucleus (**A**), cortical nucleus (**B**) and PHA (**C**) across donors. Bubble size represents correlation strength, whereas color indicates the direction (red = positive and blue = negative). *P* values are FDR-corrected. *Means *p* < 0.05, **means *p* < 0.01 and ***means *p* < 0.001. aSyn: alpha-synuclein; CDR: clinical dementia rating; LB: Lewy body; PHA: parahippocampal-amygdaloid transition area; pS129: phosphorylated serine 129

Lastly, we explored whether aSyn pathology in the PHA, basal nucleus and cortical nucleus related with clinical characteristics and neuropsychiatric symptoms. 5G4-positive aSyn pathology in the basal nucleus was negatively associated with disease duration (*r* = − 0.38, FDR-corrected *p* = 0.039) (Fig. 5A). There was a positive correlation between both 5G4-positive aSyn pathology and astrocytic aSyn inclusions in the basal nucleus and anxiety (*r* ≥ 0.35, FDR-corrected *p* = 0.039 and *p* = 0.031, respectively). pS129-aSyn pathology, LB density, 5G4-positive aSyn pathology and astrocytic aSyn inclusions in the basal and cortical nucleus, as well as pS129-aSyn pathology in the PHA, were all positively correlated with dementia (*r* ≥ 0.36, FDR-corrected *p* = 0.047 to *p* = 0.001) (Fig. 5A–C). Contrastingly, none of the mentioned aSyn pathologies in any of the hotspot nuclei correlated significantly with CDR. Also for depression no significant correlations were observed. However, pS129-aSyn pathology and LB density in the basal and cortical nucleus, and LB density in the PHA correlated positively with the presence of hallucinations (*r* ≥ 0.37, FDR-corrected *p* = 0.008 to *p* = 0.094).

## Discussion

In this study, we examined differences in the vulnerability of amygdalar nuclei to aSyn, Aβ, p-tau, neuritic plaques and p-TDP-43 pathology across multiple LBDs. Our results show that the PHA and basal nucleus were both selectively vulnerable to pS129-aSyn and 5G4-positive aSyn pathology, whereas the pattern of nuclei vulnerability to Aβ and p-tau pathology was LBD-specific. However, overall, the nuclei in the ventromedial part of the amygdala were hotspots for multiple types of protein aggregation across LBDs and aSyn pathological burden in these nuclei correlated with the presence of cognitive and neuropsychiatric symptoms.

The PHA showed the highest vulnerability to pS129-aSyn pathology in PD, DLB and AD + LB and 5G4-positive aSyn pathology in PD and DLB. However, the basal nucleus was the most vulnerable to 5G4-positive aSyn pathology in AD + LB. The LB density was highest in the PHA in PD and in the basal nucleus in DLB and AD + LB. These findings correspond to a study by Braak et al. [9] and Harding et al. [22]. The latter observed the highest LB concentration in the basal nucleus of PD donors but did not include the PHA. In contrast to our findings, Sahin and colleagues reported that the cortical nucleus displayed the highest aSyn burden in DLB [51]. This discrepancy could be due to a higher age at death (81.7 ± 9 vs. 70 ± 6.2) or differences in post-mortem delay, Braak stages, antibody use and/or nuclei delineation. Since the PHA is poorly innervated by the olfactory bulb [62], it is not likely that the high burden of both types of aSyn pathology observed here has spread from there, as predicted by the brain-first model [7]. However, the PHA contains many dense tyrosine hydroxylase-immunoreactive fibers, that are indicative of dopaminergic input [62], from the substantia nigra and ventral tegmental area [49]. LB formation in the PHA could therefore be a result of dopaminergic loss, which is characteristic of PD as well as AD [38, 47].

It has been shown before that astrocytic aSyn pathology is abundant in the amygdala in LBD [42, 54]. Astrocytic aSyn inclusions were more prevalent in DLB and AD + LB cases compared to iLBD and PD cases. This is in line with existing literature in which astrocytic aSyn pathology was more common in cases with cognitive impairment compared to without [42]. Spider, star-like and spiky astrocytic aSyn inclusions were more frequently detected in the PHA and basal nucleus compared to other nuclei. These are also the nuclei displaying the most neuronal aSyn pathology. Such distribution could be explained by the staging model proposed by Otero-Jimenez, in which astrocytic aSyn pathology in the amygdala appears once a threshold for neuronal aSyn pathology has been reached [42]. This would imply that the observed astrocytic involvement in the PHA and basal nucleus does not reflect specific vulnerability to astrocytic aSyn but rather a secondary response to neuronal involvement.

Among iLBD cases, the central nucleus was most vulnerable towards Aβ pathology. However, no plaques had been detected in this nucleus, which suggests that it was affected by other Aβ pathological structures than plaques, i.e. intracellular or intravascular immunoreactivity. The finding that the accessory basal nucleus displayed the highest Aβ pathology among DLB donors corresponds with previous post-mortem human brain research [51], but was not statistically significant in our study. In AD + LB, the PHA was the most vulnerable, followed by the cortical nucleus. It was shown before that the cortical nucleus was most affected by Aβ pathology in AD [20]. Interestingly, Aβ deposition has been associated with olfactory impairment in AD [61].

Diffuse plaques were more commonly observed than cored plaques, and their highest density was found in the accessory basal and basal nuclei of AD + LB cases. As diffuse plaques are thought to represent an earlier stage of Aβ aggregation [25], these nuclei might be preferential sites for early Aβ deposition. For cored plaques, there was no statistical significant difference in nuclei vulnerability.

P-tau pathology vulnerability was highest in the cortical nucleus in the PD group, in the PHA in the DLB group and in the basal nucleus in the AD + LB group. Existing literature reported the central nucleus to be most affected by p-tau pathology in DLB and the cortical nucleus in AD [20, 51]. However, the central nucleus of DLB cases was the least vulnerable in our study. The cortical nucleus was also one of the least affected nuclei among AD + LB cases, although it did display a relative high susceptibility to fine granular cytoplasmic neuronal tau and NFTs. This discrepancy could be assigned to non-overlapping segmentation of the cortical, medial and central nucleus [51] or to the use of a different antibody [20]. Moreover, it may also mean that amygdala nuclei vulnerability of pure AD cases is different from that of AD + LB cases.

While the basal and accessory basal nucleus of AD + LB cases were both vulnerable to p-tau pathology, the accessory basal nucleus showed a higher neurofibrillary tangle density and lower fine granular cytoplasmic neuronal tau inclusion density and the basal nucleus the other way around. This might indicate that the process of aggregation of p-tau into NFTs [56] was more prominent in the accessory basal nucleus than in the basal nucleus, but no significant differences were observed. Although not statistically significant, the highest density of neuritic plaques was detected in the basal nucleus of AD + LB donors, which is both in line and in contrast with previous studies [11, 28].

The PHA, ventral part of the basal nucleus and cortical nucleus were most often identified as hotspots for the different types of protein pathology. Thus, despite differences in amygdalar nuclei vulnerability between protein aggregates and LBDs, our results also indicate that some nuclei are common protein pathology hotspots. Specifically the ventral part of the basal nucleus is strongly connected with the entorhinal-hippocampal system [17, 62]. Whereas Aβ and p-tau pathology in this portion of the basal nucleus might have spread from the hippocampus, which is affected early and heavily in AD [8], aSyn pathology could have arisen there, as is the case in brain-first subtypes [7]. Unlike the PHA, the cortical nucleus is innervated directly by the olfactory bulb [6, 41], which is affected in early stages of AD [14, 16]. Therefore, it could be that AD-related pathology (Aβ and p-tau) in the cortical nucleus has propagated from there. For almost all types of protein pathology and morphological structures studied, the medial nucleus was the least vulnerable. The relative sparing of this nucleus could mean that the GABAergic neurons they predominantly consist of [50] are less prone to protein aggregation. Indeed, these neurons were found to be resistant to LB pathology before [55].

Protein aggregation in the amygdala may contribute to cognitive and neuropsychiatric symptoms in LBD [39]. We found that aSyn pathology in the basal nucleus positively correlated with the presence of dementia, hallucinations and anxiety and negatively with disease duration. An association between aSyn pathology burden in the basal nucleus and anxiety was previously observed in a PD mouse model and hypothesized to be due to propagation of aSyn pathology from the basal nucleus to other limbic structures [31]. Harding and colleagues also showed a positive association between LB density in the basal nucleus and the experience of visual hallucinations in PD patients [22]. In our study, this relationship existed across all LBD groups, suggesting that it may reflect a link between basal nucleus vulnerability and visual hallucinations regardless of the type of LBD. However, pS129-aSyn pathology in the cortical nucleus was also positively correlated with hallucinations across LBDs in our study, while Sekiya et al. showed that there was no significant difference in pS129-aSyn pathology in the more medial part of the amygdala between DLB cases with and without visual hallucinations [52]. This discrepancy could indicate that our correlation between aSyn pathology in the cortical nucleus and hallucinations was mainly driven by the other LBD groups. Furthermore, dementia positively correlated with aSyn pathology in all nuclei. Despite being a more specific measurement for dementia, no statistically significant correlations were observed for CDR. This could be due to lack of statistical power or could mean that it is more related to having dementia regardless of its severity. Thus, the presence of dementia might be a consequence of vulnerability of the amygdala as a whole and/or its impaired connections with other (limbic) brain structures, whereas anxiety and hallucinations could be resulting from vulnerability of the basal and cortical nucleus specifically. This could be further investigated using magnetic resonance imaging in large well-characterized clinical cohorts [29].

One of the strengths of this study is the detailed pathological characterization of the included donors. More importantly, we extend earlier work by determining and comparing the vulnerability of seven amygdalar nuclei to six types of protein pathology across multiple LBDs. In addition to total pathology burden, we quantified differences in vulnerability to specific morphological structures. To our knowledge, this is the only study that assessed protein pathology hotspots per LBD case. However, the study also has some limitations. First of all, group sizes were unequal and relatively small, thereby limiting statistical power. Secondly, only one tissue section per case per staining was used. This originated mostly from the middle of the amygdala, which might have different amygdalar nuclei vulnerability patterns then the (more) anterior or posterior part of the amygdala. Moreover, not all nuclei were present in all tissue sections, due to atrophy. To gain more insight in the variability and timing of protein aggregation in LBD, it would be interesting to include more iLBD cases with different Braak LB stages as well as more brain-first and body-first LBD cases in future studies [7]. Furthermore, by using serial tissue sections per immunostaining, covering the full anatomical range of the amygdala, a 3D reconstruction of protein pathology across the amygdalar nuclei could be created. This may aid to understand the differences in vulnerability between the more anterior and posterior nuclei within LBDs.

## Conclusions

In conclusion, our results show that the PHA, ventral part of the basal nucleus and cortical nucleus are hotspots for protein aggregation in LBDs but also that amygdala nuclei vulnerability is pathology- and LBD-specific. Together, this study provides a deeper insight into the selective vulnerability of amygdalar nuclei to protein aggregates in LBDs and their relation to clinical characteristics.

## Supplementary Information

Additional file1 (DOCX 137 KB)

Supplementary Figure 1. Manual segmentation of amygdalar nuclei in QuPath. A, C and E show pictures of plate 23 (A), 26 (C) and 28 (E) from the Atlas of the Human Brain (Mai, Assheuer and Paxinos, second edition (2004), whereas B, D and F show manual segmentation of the whole amygdala and its main nuclei in the hematoxylin channel, according to A, C and E, respectively. AB: accessory basal nucleus; Ba: basal nucleus; Ce: central nucleus; Co: cortical nucleus; La: lateral nucleus; Me: medial nucleus; PHA: parahippocampal-amygdaloid transition area.

Supplementary Figure 2. Detection of immunoreactivity by in-house developed pixel classifiers in QuPath. Representative images of immunostainings of pS129-aSyn (EP1536Y) (A), aggregated aSyn (5G4) (C), Aβ (4G8) (E), p-tau (AT8) (G) and p-TDP-43 (11-9) (I). Corresponding detections of immunoreactivity by in-house developed QuPath pixel classifiers are shown in dark red (B, D, F, H, J). Scale bar represents 100 µm. Aβ: amyloid beta; pS129-aSyn: serine 129-phosphorylated alpha-synuclein; p-tau: phosphorylated tau; p-TDP-43: phosphorylated transactive response DNA-binding protein-43.

Supplementary figure 3. Hotspot analysis. Example immunostaining of pS129-aSyn (EP1536Y) displaying a dorsal-to-ventral gradient of immunoreactivity within the basal nucleus (Ba) (A). Scan of a pS129-aSyn (EP1536Y)-stained and segmented amygdala overlaid with a grid of tiles of 25.000 µm2 each in QuPath (B). The yellow-outlined tile displays the highest EP1536Y immunoreactivity and the nucleus containing this tile (the parahippocampal-amygdaloid transition area (outlined in cyan), in this example) therefore was defined as the pS129-aSyn hotspot for this case.

Supplementary figure 4. Ordinal scoring of astrocytic aSyn severity. Example images for each score of astrocytic aSyn severity, ranging from 0 to 3, which corresponds to the detection of no, 1 to 5, 6 to 15 or more than 15 spider, spiky or star-like 5G4-positive astrocytic aSyn inclusions (Otero-Jimenez, Wojewska [42]), respectively. Red asterisks indicate (some of the) spider, spiky or star-like astrocytic aSyn inclusions. Note that the number detections is counted in annotations of 250.000 µm2, while the images shown here are 62.500 µm2. Scale bar represents 50 µm.

## Data Availability

Supporting data could be made available upon reasonable request.

## Abbreviations

Aβ: Amyloid beta
AD: Alzheimer’s disease
AD + LB: Alzheimer’s disease with Lewy bodies
aSyn: Alpha-synuclein
DLB: Dementia with Lewy bodies
iLBD: Incidental Lewy body disease
LB: Lewy body
LBD: Lewy body disease
NFT: Neurofibrillary tangle
PD: Parkinson’s disease
PHA: Parahippocampal-amygdaloid transition area
pS129: Phosphorylated serine 129
p-tau: Phosphorylated tau
p-TDP-43: Phosphorylated transactive response DNA-binding protein-43
