# Supplementary material for: Amygdalar nuclei vulnerability to protein aggregates in Lewy body diseases
Source: Acta Neuropathol Commun. 2026 Jul 21;14:216. doi: 10.1186/s40478-026-02381-0 (PMC13670467; doi:10.1186/s40478-026-02381-0)
Supplement: Supplementary file 1 [file 40478_2026_2381_MOESM1_ESM.docx]

**Supplementary table 1.** ***Definitions of clinical and neuropsychiatric characteristics***

| **Characteristic type** | **Characteristic** | **Definition** |
| --- | --- | --- |
| Clinical | Disease duration | Time in years between onset of symptoms of neurodegenerative disease and death |
|  | Parkinsonism | Bradykinesia plus rigidity and/or resting tremor [1] |
|  | Dementia | Disturbances in at least two cognitive domains or MMSE <20 at time of death |
|  | CDR | Clinical Dementia Rating, based on scores in six cognitive categories, as described in [2] |
| Neuropsychiatric | Hallucinations | Mentioning of “hallucinations” (visual and/or auditory) in the medical record |
|  | Anxiety | Mentioning of “anxiety”, “anxious” or “fear” in the medical record |
|  | Depression | Mentioning of “depression", "depressed" or "depressive” in the medical record |

**Supplementary table 2.** ***Specifications of immunohistochemistry procedure and used antibodies***

| **Epitope** | **Clone** | **Brand** | **Article nr.** | **Lot nr.** | **Species** | **HIER** | **Block 1 solution  (duration)** | **Block 2 solution (duration)** | **Dilution** | **DAB incubi-tion** |
| --- | --- | --- | --- | --- | --- | --- | --- | --- | --- | --- |
| aSyn, aggregated | 5G4 | Millipore | MABN389 | VP2206085;  VP2408086 | mouse | 10mM Tris-EDTA buffer (pH 9.0) (30 min at ±100⁰C) | TBS + 1% H_2_O_2_ (30 min) | TBS + 3% NGS (30 min) | 1:2000 in TBS + 0.5% Tx + 1% NGS | 10 min |
| aSyn, pSer129 | EP1536Y | Abcam | ab51253 | GR3437967-3 | rabbit |  |  | TBS + 0.5% Tx + 3% NGS (30 min) | 1:8000 in TBS + 0.5% Tx + 1% NGS |  |
| TDP-43, pSer409/410 | 11-9 | Cosmo Bio Co | CAC-TIP-PTD-M01A | 19-33-1 | mouse | 10mM citrate buffer  (pH 6.0)  (30 min at ±100⁰C) |  | TBS + 3% NGS (20 min) | 1:4000 in TBS + 0.5% Tx + 1% NGS |  |
| Aβ, aa 17-24 | 4G8 | BioLegend | 800708 | B374107 | mouse |  |  | TBS + 0.1% Tx + 1% NGS (30 min) | 1:4000 in TBS + 0.1% Tx + 1% NGS | 15 min |
| Tau, pSer202/pThr208 | AT8 | Thermo Scientific | MN1020 | WB3172201 | mouse |  | TBS + 0.3% H_2_O_2_ (30 min) | TBS + 0.1% Tx + 2% NGS (30 min) | 1:800 in TBS + 0.1% Tx + 2% NGS |  |

*Aβ: amyloid-beta; aa: amino acids; aSyn: alpha-synuclein; DAB: 3.3’-Diaminobenzidine; EDTA: ethylenediaminetetraacetic acid; HIER: heat-induced epitope retrieval; mM: millimolar; NGS: normal goat serum; pThr: phosphorylated threonine; pSer: phosphorylated serine; TBS: Tris-buffered saline; TDP-43: transactive response DNA-binding protein-43; Tx: Triton X-100*

**Supplementary table 3.** ***Availability of amygdalar nuclei annotations per section and donor***

| **Donor ID** | **aSyn (EP1536Y)** | **aSyn (5G4)** | **Aβ (4G8)** | **p-tau (AT8)** | **Gallyas** | **p-TDP-43 (11-9)** |
| --- | --- | --- | --- | --- | --- | --- |
| iLBD01 | AB, Ba, Ce, Co, La, Me, PHA | AB, Ba, Ce, Co, La, Me, PHA | AB, Ba, Ce, Co, La, Me, PHA | AB, Ba, Ce, Co, La, Me, PHA | AB, Ba, Ce, Co, La, Me, PHA | AB, Ba, Ce, Co, La, Me, PHA |
| iLBD02 | Ba, La | Ba, La | Ba, La | Ba, La | Ba, La | Ba, La |
| iLBD03 | AB, Ba, Ce, Co, La, Me, PHA | AB, Ba, Ce, Co, La, Me, PHA | AB, Ba, Ce, La, Me, PHA | AB, Ba, Ce, Co, La, Me, PHA | AB, Ba, Ce, Co, La, Me, PHA | AB, Ba, Ce, Co, La, Me, PHA |
| iLBD04 | AB, Ba, Ce, Co, La, Me, PHA | AB, Ba, Ce, Co, La, Me, PHA | AB, Ba, Ce, Co, La, Me, PHA | AB, Ba, Ce, Co, La, Me, PHA | AB, Ba, Ce, Co, La, Me, PHA | AB, Ba, Ce, Co, La, Me |
| iLBD05 | AB, Ba, Co, La, PHA | AB, Ba, Co, La, PHA | Ba, La | AB, Ba, Co, La, PHA | - | AB, Ba, Co, La, PHA |
| iLBD06 | AB, Ba, Ce, Co, La | AB, Ba, Ce, Co, La | AB, Ba, Ce, Co, La | AB, Ba, Ce, Co, La | AB, Ba, Ce, Co, La | AB, Ba, Ce, Co, La |
| PDD01 | AB, Ba, Ce, Co, La, Me, PHA | AB, Ba, Ce, Co, La, Me, PHA | AB, Ba, Ce, Co, La, Me, PHA | AB, Ba, Ce, Co, La, Me, PHA | AB, Ba, Ce, Co, La, Me, PHA | AB, Ba, Ce, Co, La, Me, PHA |
| PDD02 | AB, Ba, Ce, La, PHA | AB, Ba, Ce, Co, La, PHA | AB, Ba, Ce, Co, La, Me, PHA | AB, Ba, Ce, Co, La, Me, PHA | AB, Ba, Ce, Co, La, Me, PHA | AB, Ba, Ce, Co, La, Me, PHA |
| PDD03 | AB, Ba, Ce, Co, La, Me, PHA | AB, Ba, Ce, Co, La, Me, PHA | AB, Ba, Ce, Co, La, Me, PHA | AB, Ba, Ce, Co, La, Me, PHA | - | - |
| PDD04 | - | AB, Ba, Ce, Co, La, Me, PHA | AB, Ba, Ce, Co, La, Me, PHA | AB, Ba, Ce, Co, La, Me, PHA | - | - |
| PDD05 | AB, Ba, Ce, Co, La, Me, PHA | AB, Ba, Ce, Co, La, Me, PHA | AB, Ba, Ce, Co, La, Me, PHA | AB, Ba, Ce, Co, La, Me, PHA | - | - |
| PDD06 | - | AB, Ba, Ce, Co, La, Me, PHA | AB, Ba, Co, La, Me, PHA | AB, Ba, Ce, Co, La, PHA | - | - |
| PDD07 | AB, Ba, Ce, Co, La, Me, PHA | AB, Ba, Ce, Co, La, Me, PHA | AB, Ba, Ce, Co, La, Me, PHA | AB, Ba, Ce, Co, La, Me, PHA | - | - |
| PDD08 | - | AB, Ba, Co, La, PHA | AB, Ba, Ce, Co, La, PHA | AB, Ba, Co, La, PHA | - | - |
| PDD09 | AB, Ba, Ce, Co, La, Me, PHA | AB, Ba, Ce, Co, La, Me, PHA | AB, Ba, Ce, Co, La, Me, PHA | AB, Ba, Ce, Co, La, Me, PHA | AB, Ba, Ce, Co, La, Me, PHA | AB, Ba, Ce, Co, La, Me, PHA |
| PDD10 | AB, Ba, Ce, Co, La, Me, PHA | AB, Ba, Ce, Co, La | AB, Ba, Ce, Co, La, Me, PHA | AB, Ba, Ce, Co, La, Me, PHA | - | - |
| PDD11 | AB, Ba, Ce, La, Me, PHA | AB, Ba, Ce, La, Me, PHA | AB, Ba, Ce, Co, La, Me, PHA | AB, Ba, Ce, Co, La, Me, PHA | - | - |
| PDD12 | AB, Ba, Ce, Co, La | AB, Ba, Ce, Co, La | AB, Ba, Ce, Co, La | AB, Ba, Ce, Co, La | - | - |
| PDD13 | AB, Ba, Ce, Co, La, Me, PHA | AB, Ba, Ce, Co, La, Me, PHA | - | - | AB, Ba, Ce, Co, La, Me, PHA | AB, Ba, Ce, Co, La, Me |
| PDD14 | AB, Ba, Ce, Co, La, Me, PHA | AB, Ba, Ce, Co, La, Me, PHA | AB, Ba, Ce, Co, La, Me, PHA | AB, Ba, Ce, Co, La, Me, PHA | AB, Ba, Ce, Co, La, Me, PHA | AB, Ba, Ce, Co, La, Me, PHA |
| PDD15 | AB, Ba, Ce, Co, La, Me, PHA | AB, Ba, Ce, Co, La, Me, PHA | AB, Ba, Ce, Co, La, Me, PHA | AB, Ba, Ce, Co, La, Me, PHA | AB, Ba, Ce, Co, La, Me, PHA | AB, Ba, Ce, Co, La, Me, PHA |
| PDD16 | AB, Ba, Ce, Co, La | AB, Ba, Ce, Co, La | AB, Ba, Ce, Co, La | AB, Ba, Ce, Co, La | AB, Ba, Ce, Co, La | AB, Ba, Ce, Co, La |
| PDD17 | AB, Ba, Ce, Co, La, Me, PHA | AB, Ce, Co, La, PHA | AB, Ba, Ce, La | AB, Ba, Ce, Co, La, Me, PHA | AB, Ba, Ce, Co, La, Me, PHA | AB, Ba, Ce, Co, La, PHA |
| PDD18 | AB, Ba, Ce, Co, La, Me, PHA | AB, Ba, Ce, Co, La, Me, PHA | AB, Ba, Ce, La | AB, Ba, Ce, Co, La, Me, PHA | AB, Ba, Ce, Co, La, Me, PHA | AB, Ba, Ce, Co, La, Me, PHA |
| DLB01 | AB, Ba, Ce, Co, La, Me, PHA | AB, Ba, Ce, Co, La, Me, PHA | AB, Ba, Ce, Co, La, Me, PHA | AB, Ba, Ce, Co, La, Me, PHA | AB, Ba, Ce, Co, La, Me, PHA | AB, Ba, Ce, Co, La, Me, PHA |
| DLB02 | AB, Ba, Ce, Co, La, Me, PHA | AB, Ba, Ce, Co, La, Me, PHA | AB, Ba, Ce, Co, La, Me, PHA | AB, Ba, Ce, Co, La, Me, PHA | AB, Ba, Ce, Co, La, Me, PHA | AB, Ba, Ce, Co, La, Me, PHA |
| DLB03 | AB, Ba, Ce, Co, La, Me, PHA | AB, Ba, Ce, Co, La, Me, PHA | AB, Ba, Ce, Co, La, Me, PHA | AB, Ba, Ce, Co, La, Me, PHA | AB, Ba, Ce, Co, La, Me, PHA | AB, Ba, Ce, Co, La, Me, PHA |
| DLB04 | AB, Ba, Ce, Co, La, Me | AB, Ba, Ce, Co, La, Me, PHA | AB, Ba, Ce, Co, La, Me, PHA | AB, Ba, Ce, Co, La, Me, PHA | AB, Ba, Ce, Co, La, Me, PHA | AB, Ba, Ce, Co, La, Me, PHA |
| DLB05 | AB, Ba, La | AB, Ba, La | AB, Ba, La | AB, Ba, La | AB, Ba, La | AB, Ba, La |
| DLB06 | AB, Ba, Ce, Co, La, Me, PHA | AB, Ba, Ce, Co, La, Me, PHA | AB, Ba, Ce, Co, La, Me, PHA | AB, Ba, Ce, Co, La, Me, PHA | AB, Ba, Ce, Co, La, Me, PHA | AB, Ba, Ce, Co, La, Me, PHA |
| DLB07 | AB, Ba, Ce, Co, La, Me, PHA | AB, Ba, Ce, Co, La, PHA | AB, Ba, Ce, Co, La | AB, Ba, Ce, Co, La, Me, PHA | - | - |
| DLB08 | AB, Ba, Ce, Co, La, Me, PHA | AB, Ba, Ce, Co, La, Me, PHA | - | AB, Ba, Ce, Co, La, Me, PHA | - | - |
| DLB09 | AB, Ba, Ce, Co, La, Me, PHA | AB, Ba, Ce, Co, La, Me, PHA | AB, Ba, Ce, Co, La, Me, PHA | AB, Ba, Ce, Co, La, Me, PHA | AB, Ba, Ce, Co, La, Me, PHA | AB, Ba, Ce, Co, La, Me, PHA |
| ADLB01 | AB, Ba, Ce, Co, La, Me, PHA | AB, Ba, Ce, Co, La, Me, PHA | AB, Ba, Ce, Co, La, Me, PHA | AB, Ba, Ce, Co, La, Me, PHA | AB, Ba, Ce, Co, La, Me, PHA | AB, Ba, Ce, Co, La, Me, PHA |
| ADLB02 | AB, Ba, Ce, Co, La | AB, Ba, Ce, Co, La | AB, Ba, Ce, Co, La | AB, Ba, Ce, Co, La | AB, Ba, Ce, Co, La | AB, Ba, Ce, Co, La |
| ADLB03 | AB, Ba, Ce, Co, La | AB, Ba, Ce, Co, La | AB, Ba, Ce, Co, La | AB, Ba, Ce, Co, La | AB, Ba, Ce, Co, La | AB, Ba, Ce, Co, La |
| ADLB04 | AB, Ba, Ce, Co, La | AB, Ba, Ce, Co, La | AB, Ba, Ce, Co, La | AB, Ba, Ce, Co, La | AB, Ba, Ce, Co, La | AB, Ba, Ce, Co, La |
| ADLB05 | AB, Ba, Ce, Co, La, Me, PHA | AB, Ba, Ce, Co, La, Me, PHA | AB, Ba, Ce, Co, La, Me, PHA | AB, Ba, Ce, Co, La, PHA | AB, Ba, Ce, Co, La, Me, PHA | AB, Ba, Ce, Co, La, Me, PHA |
| ADLB06 | AB, Ba, Ce, Co, La, Me, PHA | AB, Ba, Ce, Co, La, Me, PHA | AB, Ba, Ce, Co, La, Me, PHA | AB, Ba, Ce, Co, La, Me, PHA | AB, Ba, Ce, Co, La, Me, PHA | AB, Ba, Ce, Co, La, Me, PHA |
| ADLB07 | AB, Ba, Ce, Co, La, PHA | AB, Ba, Ce, Co, La, PHA | AB, Ba, Ce, Co, La, PHA | AB, Ba, Ce, Co, La, PHA | AB, Ba, Ce, Co, La, PHA | AB, Ba, Ce, Co, La, PHA |
| ADLB08 | AB, Ba, Ce, Co, La | AB, Ba, Ce, Co, La | AB, Ba, La | AB, Ba, Ce, Co, La | AB, Ba, Ce, Co, La | AB, Ba, Ce, Co, La |
| ADLB09 | AB, Ba, Ce, Co, La, Me, PHA | AB, Ba, Ce, Co, La, Me, PHA | AB, Ba, Ce, Co, La, Me, PHA | AB, Ba, Ce, Co, La, Me, PHA | - | - |
| ADLB10 | AB, Ba, Ce, Co, La, Me, PHA | AB, Ba, Ce, Co, La, Me, PHA | AB, Ba, Ce, Co, La, Me, PHA | AB, Ba, Ce, Co, La, Me, PHA | - | - |
| ADLB11 | AB, Ba, Ce, Co, La | AB, Ba, Ce, Co, La | AB, Ba, Ce, Co, La | AB, Ba, Ce, Co, La | AB, Ba, Ce, Co, La | AB, Ba, Ce, Co, La |
| ADLB12 | AB, Ba, Ce, Co, La, Me, PHA | AB, Ba, Ce, Co, La, Me, PHA | AB, Ba, Ce, Co, La, Me, PHA | AB, Ba, Ce, Co, La, Me, PHA | - | - |
| ADLB13 | AB, Ba, Ce, Co, La, Me, PHA | AB, Ba, Ce, Co, La, Me, PHA | AB, Ba, Ce, Co, La, Me, PHA | AB, Ba, Ce, Co, La, Me, PHA | - | - |
| ADLB14 | AB, Ba, Ce, Co, La | AB, Ba, Ce, Co, La | AB, Ba, Ce, Co, La | AB, Ba, Ce, Co, La |  |  |
| ADLB15 | AB, Ba, Ce, Co, La, PHA | AB, Ba, Ce, Co, La, Me, PHA | AB, Ba, Ce, Co, La, PHA | AB, Ba, Ce, Co, La, PHA | AB, Ba, Ce, Co, La, Me, PHA | AB, Ba, Ce, Co, La, Me, PHA |

**Supplementary table 4.** ***Specifications for analysis of neuritic plaques in Gallyas-stained sections***

| **Nucleus size** | **Number of tiles of 640,000 µm²** |
| --- | --- |
| > 8,000,000 µm² | 2 tiles |
| > 16,000,000 µm² | 3 tiles |
| > 24,000,000 µm² | 4 tiles |
| > 32,000,000 µm² | 5 tiles |

**Supplementary table 5.** ***Definitions and outcome measurements used fur morphological analyses***

| **Immunostaining (antibody clone)** | **morphological structure** | **definition** | | **outcome measurement** |
| --- | --- | --- | --- | --- |
|  |  | ***shape*** | ***diameter*** |  |
| pS129-aSyn (EP1536Y) | Lewy body | Spherical [3] or ring-shaped [4] IR in the cytoplasm of a neuron | > 7 µm* | absolute density per 0.25 mm^2^ |
| aggregated aSyn (5G4) | astrocytic inclusion | Spider, star-like or spiky immunoreactive extensions around a glial nucleus [5] | 5-50 µm | ordinal score |
| p-tau (AT8) | fine granular cytoplasmic IR | Fine granular staining of the neuronal cytoplasm and processes [6] | not specified | absolute density per 0.25 mm^2^ |
|  | neurofibrillary tangle | Dense, globose or flame-shaped neuronal cytoplasmic staining [6] | > 10 µm* | absolute density per 0.25 mm^2^ |
| Aβ (4G8) | diffuse plaque | Extracellular amorphous and irregular [7] IR with ill-defined margins [8] | > 10 µm | absolute density per 0.64 mm^2^ |
|  | cored plaque | Extracellular IR consisting of a dense central core and an outer, more diffuse corona [9] | 10 – 200 µm | absolute density per 0.64 mm^2^ |
| p-TDP-43 (11-9) | neuronal cytoplasmic inclusion [10] | Globose, spherical or tangle-like [10] IR in the cytoplasm of a neuron | > 5 µm* | absolute density per 0.25 mm^2^ |
|  | process [11] | Long or short thin neurite-like IR | not specified | absolute density per 0.25 mm^2^ |
| Gallyas | neuritic plaque | Cluster of punctate, bulbous and thread-like structures [12] containing minimal three neuritic threads or dystrophic neurites. | > 30 µm | absolute density per 0.64 mm^2^ |

* when the nucleus is not visible, i.e. if the inclusion overlies the nucleus.

*IR: immunoreactivity*

**Supplementary table 6.** ***Clinical characteristics of included donors***

| **Donor ID** | **Age at symptom onset** (years) | **Disease duration** (years) | **Parkinsonism** yes/no | **Dementia** yes/no | **Hallucinations** yes/no | **Anxiety** yes/no | **Depression** yes/no |
| --- | --- | --- | --- | --- | --- | --- | --- |
| iLBD01 | NA | NA | no | no | no | unknown | 1 |
| iLBD02 | NA | NA | no | no | no | 0 | 0 |
| iLBD03 | NA | NA | no | no | no | 0 | 0 |
| iLBD04 | NA | NA | no | no | no | 0 | 0 |
| iLBD05 | NA | NA | no | no | no | 0 | 0 |
| iLBD06 | NA | NA | no | no | no | 1 | 1 |
| PD01 | 55 | 11 | yes | yes | no | 0 | 0 |
| PD02 | 61 | 13 | yes | no | yes | 1 | 1 |
| PD03 | 69 | 6 | yes | no | yes | 0 | 1 |
| PD04 | 81 | 7 | yes | yes | yes | 0 | 0 |
| PD05 | 60 | 21 | yes | yes | yes | 1 | 1 |
| PD06 | 49 | 11 | yes | yes | yes | 1 | 1 |
| PD07 | 65 | 6 | yes | yes | yes | 0 | 0 |
| PD08 | 76 | 8 | yes | yes | yes | 0 | 0 |
| PD09 | 64 | 6 | yes | yes | yes | 0 | 0 |
| PD10 | 53 | 19 | yes | no | yes | 0 | 0 |
| PD11 | 63 | 17 | yes | yes | yes | 1 | 0 |
| PD12 | 72 | 17 | yes | yes | yes | 1 | 1 |
| PD13 | 60 | 22 | yes | no | yes | 0 | 1 |
| PD14 | 68 | 9 | yes | no | no | 1 | 0 |
| PD15 | ≤55 | ≥20 | yes | no | yes | 0 | 0 |
| PD16 | 49 | 28 | yes | no | yes | 0 | 0 |
| PD17 | 41 | 32 | yes | yes | yes | 0 | 1 |
| PD18 | 76 | 12 | yes | yes | yes | 1 | 1 |
| DLB01 | 66 | 8 | yes | yes | yes | 0 | 0 |
| DLB02 | 54 | 4 | yes | yes | yes | 1 | 1 |
| DLB03 | 70 | 7 | no | yes | yes | 0 | 1 |
| DLB04 | 61 | 6 | yes | yes | yes | 1 | 1 |
| DLB05 | 62 | 8 | yes | yes | yes | 1 | 1 |
| DLB06 | 52 | 15 | yes | yes | yes | 1 | 1 |
| DLB07 | 66 | 4 | yes | yes | yes | 1 | 1 |
| DLB08 | 61 | 7 | yes | yes | yes | 1 | 1 |
| DLB09 | 74 | 5 | yes | yes | yes | 1 | 1 |
| ADLB01 | 72 | 9 | no | yes | no | 1 | 1 |
| ADLB02 | 51 | 16 | yes | yes | yes | 0 | 0 |
| ADLB03 | 74 | 5 | no | yes | yes | 1 | 1 |
| ADLB04 | 75 | 6 | yes | yes | yes | 1 | 1 |
| ADLB05 | 66 | 7 | yes | yes | no | 0 | 0 |
| ADLB06 | 62 | 5 | yes | yes | yes | 1 | 1 |
| ADLB07 | 49 | 7 | yes | yes | yes | 0 | 1 |
| ADLB08 | 59 | 8 | no | yes | yes | 1 | 0 |
| ADLB09 | 63 | 7 | no | yes | no | 0 | 1 |
| ADLB10 | 67 | 6 | yes | yes | yes | 1 | 1 |
| ADLB11 | 57 | 6 | yes | yes | yes | 1 | 1 |
| ADLB12 | 75 | 6 | yes | yes | yes | 1 | 0 |
| ADLB13 | unknown | unknown | unknown | yes | no | unknown | unknown |
| ADLB14 | 59 | 16 | no | yes | yes | 1 | 0 |
| ADLB15 | 79 | 1 | yes | yes | yes | 0 | 0 |

**Supplementary table 7.** ***Neuropathological characteristics of included donors***

| **Donor ID** | **Sex** F/M | **Age at death**  (years) | **Cause of death** | **PMD** (h:min) | **Braak LB stage** | **Braak NFT stage** | **Thal phase** | **ABC score** | **CAA** type (stage) | **ARTAG** |
| --- | --- | --- | --- | --- | --- | --- | --- | --- | --- | --- |
| iLBD01 | M | 72 | hypovolemic shock | 8:25 | 4 | 1 | 1 | A1B1C0 | NA | no |
| iLBD02 | M | 76 | prostate carcinoma | 4:45 | 5 | 0 | 1 | A1B1 | NA | no |
| iLBD03 | M | 84 | anemia/prostate carcinoma | 7:20 | atypical | 1 | 1 | A1B1C0 | NA | no |
| iLBD04 | F | 89 | euthanasia | 4:45 | 4 | 2 | 2 | A1B1C0 | NA | no |
| iLBD05 | F | 87 | pneumonia | 7:00 | 4 | 1 | 0 | A0B0C0 | NA | no |
| iLBD06 | F | 85 | pneumonia | 6:40 | 4 | 3 | 1 | A1B2C0 | NA | no |
| PD01 | F | 66 | euthanasia | 7:10 | 5 | 1 | 3 | A2B1C0 | NA | no |
| PD02 | F | 74 | cachexia/dehydration | 3:45 | 6 | 2 | 3 | A2B1C1 | 2 (1) | no |
| PD03 | M | 75 | euthanasia | 3:35 | 6 | 1 | 1 | A1B1C0 | NA | slight |
| PD04 | F | 88 | euthanasia | 6:05 | 6 | 2 | 1 | A1B1C0 | NA | slight |
| PD05 | M | 81 | euthanasia | 6:10 | 6 | 3 | 0 | A0B2C0 | NA | slight |
| PD06 | M | 60 | euthanasia | 8:30 | 6 | 0 | 0 | A0B0C0 | NA | no |
| PD07 | M | 71 | euthanasia | 4:55 | 6 | 1 | 3 | A2B1C0 | 2 (3) | very slight |
| PD08 | M | 84 | malignancy | 4:50 | 5 | 3 | 0 | A0B2C0 | NA | slight |
| PD09 | M | 70 | reduced oral intake | 4:40 | 6 | 3 | 3 | A2B2C2 | 2 (?) | no |
| PD10 | M | 72 | dehydration | 3:50 | 6 | 1 | 1 | A1B1C0 | NA | slight |
| PD11 | M | 80 | euthanasia | 3:50 | 5 | 1 | 1 | A1B1C0 | NA | no |
| PD12 | F | 89 | cachexia/ dehydration/ dysphagia | 6:35 | 6 | 2 | 1 | A1B1C0 | NA | slight |
| PD13 | F | 82 | pneumonia | 9:17 | 6 | 2 | 2 | A1B1C0 | NA | no |
| PD14 | M | 77 | possibly cachexia/ dehydration | 6:35 | 6 | 2 | 3 | A2B1C1 | NA | slight |
| PD15 | M | 75 | pneumonia | 5:40 | 6 | 2 | 3 | A2B1C0 | 1 (?) | no |
| PD16 | F | 77 | dehydration | 5:50 | 6 | 3 | 3 | A2B2C2 | 2 (1) | no |
| PD17 | F | 73 | dehydration/hypoxia/ constipation | 4:30 | 6 | 2 | 0 | A0B1C0 | 2 (1) | no |
| PD18 | M | 88 | dehydration | 7:45 | 6 | 2 | 1 | A1B1C0 | NA | no |
| DLB01 | M | 74 | pneumonia | 4:25 | 4 | 2 | 2 | A1B2C2 | NA | no |
| DLB02 | M | 58 | hipjoint fracture | 5:25 | 6 | 1 | 1 | A1B1C0 | 2 (1) | no |
| DLB03 | M | 77 | acute myocardial infarction | 8:15 | 5 | 3 | 3 | A2B2C1 | 1 (1) | slight |
| DLB04 | F | 67 | cachexia/dehydration | 7:45 | 5 | 3 | 4 | A3B2C1 | 2 (1) | no |
| DLB05 | M | 70 | pneumonia | 7:40 | 6 | 3 | 4 | A3B2C1 | 1 (1) | yes |
| DLB06 | M | 67 | dehydration | 6:00 | 6 | 2 | 2 | A1B1C0 | NA | no |
| DLB07 | M | 70 | pneumonia | 4:45 | 6 | 1 | 1 or 2 | A1B1C0 | 2 (1) | yes |
| DLB08 | M | 68 | dehydration | 4:55 | 6 | 1 | 3 | A2B1C0 | NA | no |
| DLB09 | M | 79 | infection | 4:45 | 6 | 3 | 3 | A2B2C1 | NA | yes |
| ADLB01 | F | 81 | cachexia | 5:55 | atypical | 6 | 3 | A2B3C3 | NA | no |
| ADLB02 | M | 67 | pneumonia | 9:20 | atypical | 6 | 4 | A3B3C1 | NA | no |
| ADLB03 | F | 79 | cachexia/dehydration | 4:20 | 6 | 5 | 3 | A2B3C3 | NA | no |
| ADLB04 | F | 81 | cachexia/dehydration | 3:35 | 6 | 5 | 4 | A3B3C2 | NA | no |
| ADLB05 | M | 73 | pneumonia | 6:30 | 5 | 5 | 4 | A3B3C2 | NA | no |
| ADLB06 | M | 67 | pneumonia | 5:20 | 6 | 5 | 4 | A3B3C3 | - | yes |
| ADLB07 | M | 56 | deterioration | 5:00 | 6 | 5 | 4 | A3B3C3 | 1 (?) | no |
| ADLB08 | F | 67 | CVA | 4:30 | 4 | 6 | 4 | ? | ? | no |
| ADLB09 | M | 70 | pneumonia | 5:10 | 6 | 5 | 4 | A3B3C3 | ? (1) | no |
| ADLB10 | M | 73 | possibly DVT | 7:55 | 6 | 4 | 4 | A3B2C1 | 1 (2) | slight |
| ADLB11 | M | 63 | cachexia/dehydration | 9:50 | 6 | 6 | 5 | A3B3C3 | 2 (2) | yes |
| ADLB12 | F | 81 | dementia deterioration | 4:05 | 6 | 5 | 5 | A3B3C3 | 2 (1) | yes |
| ADLB13 | M | 77 | palliative sedation | 4:25 | 6 | 4 | 3 | A2B2C2 | 2 (1) | slight |
| ADLB14 | M | 75 | general deterioration | 6:15 | 6 | 5 | 4 | A3B3C3 | NA | no |
| ADLB15 | M | 80 | pneumonia | 5:30 | 6 | 6 | 4 or 5 | A3B3C3 | NA | no |

*AD-LB: Alzheimer’s disease with Lew bodies; ARTAG: age-related tau astrogliopathy; CAA: cerebral amyloid angiopathy; CVA: cerebrovascular accident; DLB: dementia with Lewy bodies; DVT: deep vein thrombosis; F: female; g: gram; h: hours; HS: hippocampal sclerosis; iLBD: incidental Lewy body disease; LB: Lewy body; M: male; min: minutes; NA: not applicable; NFT: neurofibrillary tangle; PD: Parkinson’s disease; PDD: Parkinson’s disease dementia; PMD: post-mortem delay; y: years; ?: unknown*

**Supplementary table 8. *Pairwise comparisons for pS129-aSyn pathology, Lewy body density and 5G4-positive aSyn pathology***

A) *pS129-aSyn pathology between nuclei within groups*

|  | ***iLBD*** | | | | | | |  | ***PD*** | | | | | | |
| --- | --- | --- | --- | --- | --- | --- | --- | --- | --- | --- | --- | --- | --- | --- | --- |
|  | **AB** | **Ba** | **Ce** | **Co** | **La** | **Me** | **PHA** |  | **AB** | **Ba** | **Ce** | **Co** | **La** | **Me** | **PHA** |
| **AB** | NA |  |  |  |  |  |  |  | NA |  |  |  |  |  |  |
| **Ba** | 0.423 | NA |  |  |  |  |  |  | **0.000** | NA |  |  |  |  |  |
| **Ce** | 0.342 | 1.000 | NA |  |  |  |  |  | **0.011** | 0.657 | NA |  |  |  |  |
| **Co** | 0.985 | 0.904 | 0.813 | NA |  |  |  |  | 1.000 | **0.000** | **0.008** | NA |  |  |  |
| **La** | 0.292 | **0.000** | **0.001** | **0.043** | NA |  |  |  | 1.000 | **0.000** | **0.008** | 1.000 | NA |  |  |
| **Me** | 0.994 | 0.217 | 0.158 | 0.823 | 0.905 | NA |  |  | 0.867 | **0.000** | **0.000** | 0.952 | 0.900 | NA |  |
| **PHA** | **0.017** | 0.715 | 0.919 | 0.136 | **0.000** | **0.008** | NA |  | **0.000** | **0.000** | **0.000** | **0.000** | **0.000** | **0.000** | NA |
|  |  |  |  |  |  |  |  |  |  |  |  |  |  |  |  |
|  | ***DLB*** | | | | | | |  | ***AD+LB*** | | | | | | |
|  | **AB** | **Ba** | **Ce** | **Co** | **La** | **Me** | **PHA** |  | **AB** | **Ba** | **Ce** | **Co** | **La** | **Me** | **PHA** |
| **AB** | NA |  |  |  |  |  |  |  | NA |  |  |  |  |  |  |
| **Ba** | 0.052 | NA |  |  |  |  |  |  | 0.057 | NA |  |  |  |  |  |
| **Ce** | 0.671 | 0.880 | NA |  |  |  |  |  | 0.919 | **0.001** | NA |  |  |  |  |
| **Co** | 0.993 | 0.322 | 0.971 | NA |  |  |  |  | 0.978 | 0.374 | 0.424 | NA |  |  |  |
| **La** | 0.949 | **0.002** | 0.131 | 0.635 | NA |  |  |  | 0.999 | 0.173 | 0.695 | 1.000 | NA |  |  |
| **Me** | **0.031** | **0.000** | **0.000** | **0.004** | 0.321 | NA |  |  | 0.071 | **0.000** | 0.433 | **0.010** | **0.026** | NA |  |
| **PHA** | **0.000** | **0.015** | **0.000** | **0.000** | **0.000** | **0.000** | NA |  | **0.000** | **0.021** | **0.000** | **0.000** | **0.000** | **0.000** | NA |

B) *LB density between nuclei within groups*

|  | ***iLBD*** | | | | | | |  | ***PD*** | | | | | | |
| --- | --- | --- | --- | --- | --- | --- | --- | --- | --- | --- | --- | --- | --- | --- | --- |
|  | **AB** | **Ba** | **Ce** | **Co** | **La** | **Me** | **PHA** |  | **AB** | **Ba** | **Ce** | **Co** | **La** | **Me** | **PHA** |
| **AB** | NA |  |  |  |  |  |  |  | NA |  |  |  |  |  |  |
| **Ba** | 0.967 | NA |  |  |  |  |  |  | **0.000** | NA |  |  |  |  |  |
| **Ce** | 0.797 | 0.998 | NA |  |  |  |  |  | 0.994 | **0.000** | NA |  |  |  |  |
| **Co** | 0.813 | 0.290 | 0.133 | NA |  |  |  |  | 0.692 | 0.064 | 0.294 | NA |  |  |  |
| **La** | 0.998 | 0.674 | 0.395 | 0.967 | NA |  |  |  | 0.999 | **0.001** | 0.898 | 0.930 | NA |  |  |
| **Me** | 0.751 | 0.476 | 0.346 | 0.991 | 0.875 | NA |  |  | **0.008** | **0.000** | 0.056 | **0.000** | **0.002** | NA |  |
| **PHA** | 0.997 | 1.000 | 0.995 | 0.545 | 0.931 | 0.575 | NA |  | **0.000** | 0.858 | **0.000** | **0.002** | **0.000** | **0.000** | NA |
|  |  |  |  |  |  |  |  |  |  |  |  |  |  |  |  |
|  | ***DLB*** | | | | | | |  | ***AD+LB*** | | | | | | |
|  | **AB** | **Ba** | **Ce** | **Co** | **La** | **Me** | **PHA** |  | **AB** | **Ba** | **Ce** | **Co** | **La** | **Me** | **PHA** |
| **AB** | NA |  |  |  |  |  |  |  | NA |  |  |  |  |  |  |
| **Ba** | **0.026** | NA |  |  |  |  |  |  | **0.000** | NA |  |  |  |  |  |
| **Ce** | 1.000 | **0.018** | NA |  |  |  |  |  | 0.955 | **0.000** | NA |  |  |  |  |
| **Co** | 0.994 | **0.003** | 0.999 | NA |  |  |  |  | 0.832 | **0.009** | 0.248 | NA |  |  |  |
| **La** | 0.260 | **0.000** | 0.410 | 0.717 | NA |  |  |  | 0.999 | **0.000** | 0.748 | 0.984 | NA |  |  |
| **Me** | **0.000** | **0.000** | **0.000** | **0.000** | **0.033** | NA |  |  | **0.000** | **0.000** | **0.001** | **0.000** | **0.000** | NA |  |
| **PHA** | 0.328 | 0.986 | 0.222 | 0.074 | **0.000** | **0.000** | NA |  | **0.018** | 0.988 | **0.001** | 0.305 | 0.067 | **0.000** | NA |

C) *5G4-positive aSyn pathology between nuclei within groups*

|  | ***iLBD*** | | | | | | |  | ***PD*** | | | | | | |
| --- | --- | --- | --- | --- | --- | --- | --- | --- | --- | --- | --- | --- | --- | --- | --- |
|  | **AB** | **Ba** | **Ce** | **Co** | **La** | **Me** | **PHA** |  | **AB** | **Ba** | **Ce** | **Co** | **La** | **Me** | **PHA** |
| **AB** | NA |  |  |  |  |  |  |  | NA |  |  |  |  |  |  |
| **Ba** | 0.195 | NA |  |  |  |  |  |  | **0.000** | NA |  |  |  |  |  |
| **Ce** | 0.748 | 0.991 | NA |  |  |  |  |  | 0.621 | **0.049** | NA |  |  |  |  |
| **Co** | 1.000 | 0.435 | 0.932 | NA |  |  |  |  | 0.998 | **0.000** | 0.294 | NA |  |  |  |
| **La** | 0.863 | 0.884 | 1.000 | 0.982 | NA |  |  |  | 1.000 | **0.000** | 0.745 | 1.000 | NA |  |  |
| **Me** | 0.978 | 0.907 | 0.999 | 0.999 | 1.000 | NA |  |  | 0.457 | **0.000** | **0.013** | 0.790 | 0.350 | NA |  |
| **PHA** | **0.046** | 0.983 | 0.794 | 0.132 | 0.492 | 0.545 | NA |  | **0.000** | **0.005** | **0.000** | **0.000** | **0.000** | **0.000** | NA |
|  |  |  |  |  |  |  |  |  |  |  |  |  |  |  |  |
|  | ***DLB*** | | | | | | |  | ***AD+LB*** | | | | | | |
|  | **AB** | **Ba** | **Ce** | **Co** | **La** | **Me** | **PHA** |  | **AB** | **Ba** | **Ce** | **Co** | **La** | **Me** | **PHA** |
| **AB** | NA |  |  |  |  |  |  |  | NA |  |  |  |  |  |  |
| **Ba** | **0.001** | NA |  |  |  |  |  |  | **0.038** | NA |  |  |  |  |  |
| **Ce** | 0.987 | **0.022** | NA |  |  |  |  |  | 0.347 | **0.000** | NA |  |  |  |  |
| **Co** | 0.083 | **0.000** | **0.010** | NA |  |  |  |  | 0.844 | **0.000** | 0.985 | NA |  |  |  |
| **La** | 0.998 | **0.006** | 1.000 | **0.019** | NA |  |  |  | 1.000 | **0.024** | 0.442 | 0.907 | NA |  |  |
| **Me** | **0.000** | **0.000** | **0.000** | 0.294 | **0.000** | NA |  |  | **0.000** | **0.000** | **0.005** | **0.000** | **0.000** | NA |  |
| **PHA** | **0.000** | **0.001** | **0.000** | **0.000** | **0.000** | **0.000** | NA |  | **0.041** | 0.999 | **0.000** | **0.001** | **0.028** | **0.000** | NA |

Post-hoc pairwise comparisons for pS129-aSyn pathology (total %area of EP1536Y immunoreactivity) (A), LB density (in number per 0.25 mm^2^) (B) and 5G4-positive aSyn pathology (total % area of 5G4 immunoreactivity) (C) between amygdalar nuclei within groups were computed using estimated marginal means with Tukey’s method for multiple comparisons. Displayed are the p-values that correspond to these adjusted pairwise contrasts, rounded to three decimals. Statistically significant (p<0.05) comparisons are indicated in bold. *AB: accessory basal nucleus; AD+LB: Alzheimer’s disease with Lewy bodies; Ba: basal nucleus; Ce: central nucleus; Co: cortical nucleus; DLB: dementia with Lewy bodies; iLBD: incidental Lewy body disease; La: lateral nucleus; Me: medial nucleus; NA: not applicable; PD: Parkinson’s disease; PHA: parahippocampal-amygdaloid transition area.*

**Supplementary table 9. *Pairwise comparisons for Aβ pathology, diffuse plaques, p-tau pathology and granular cytoplasmic neuronal tau inclusions***

A) *Aβ pathology between nuclei within groups*

|  | ***iLBD*** | | | | | | |  | ***PD*** | | | | | | |
| --- | --- | --- | --- | --- | --- | --- | --- | --- | --- | --- | --- | --- | --- | --- | --- |
|  | **AB** | **Ba** | **Ce** | **Co** | **La** | **Me** | **PHA** |  | **AB** | **Ba** | **Ce** | **Co** | **La** | **Me** | **PHA** |
| **AB** | NA |  |  |  |  |  |  |  | NA |  |  |  |  |  |  |
| **Ba** | 0.999 | NA |  |  |  |  |  |  | 0.999 | NA |  |  |  |  |  |
| **Ce** | 0.993 | 0.890 | NA |  |  |  |  |  | 1.000 | 1.000 | NA |  |  |  |  |
| **Co** | 0.919 | 0.990 | 0.580 | NA |  |  |  |  | 0.996 | 0.938 | 0.965 | NA |  |  |  |
| **La** | 0.964 | 0.998 | 0.636 | 1.000 | NA |  |  |  | 0.995 | 1.000 | 1.000 | 0.880 | NA |  |  |
| **Me** | 1.000 | 0.976 | 1.000 | 0.790 | 0.853 | NA |  |  | 1.000 | 1.000 | 1.000 | 0.995 | 0.999 | NA |  |
| **PHA** | 0.173 | 0.329 | **0.035** | 0.876 | 0.598 | 0.093 | NA |  | 1.000 | 1.000 | 1.000 | 0.994 | 0.999 | 1.000 | NA |
|  |  |  |  |  |  |  |  |  |  |  |  |  |  |  |  |
|  | ***DLB*** | | | | | | |  | ***AD+LB*** | | | | | | |
|  | **AB** | **Ba** | **Ce** | **Co** | **La** | **Me** | **PHA** |  | **AB** | **Ba** | **Ce** | **Co** | **La** | **Me** | **PHA** |
| **AB** | NA |  |  |  |  |  |  |  | NA |  |  |  |  |  |  |
| **Ba** | 0.988 | NA |  |  |  |  |  |  | 0.920 | NA |  |  |  |  |  |
| **Ce** | 0.575 | 0.946 | NA |  |  |  |  |  | 0.066 | **0.002** | NA |  |  |  |  |
| **Co** | 0.990 | 0.765 | 0.186 | NA |  |  |  |  | 0.722 | 0.999 | **0.000** | NA |  |  |  |
| **La** | 0.988 | 1.000 | 0.947 | 0.760 | NA |  |  |  | 0.386 | **0.028** | 0.977 | **0.009** | NA |  |  |
| **Me** | 0.822 | 0.994 | **1.000** | 0.404 | 0.995 | NA |  |  | 0.087 | **0.006** | 0.999 | **0.002** | 0.900 | NA |  |
| **PHA** | 0.989 | 1.000 | 0.970 | 0.790 | 1.000 | 0.997 | NA |  | **0.001** | **0.022** | **0.000** | 0.069 | **0.000** | **0.000** | NA |

B) *Diffuse plaque density between nuclei within groups*

|  | ***iLBD*** | | | | | | |  | ***PD*** | | | | | | |
| --- | --- | --- | --- | --- | --- | --- | --- | --- | --- | --- | --- | --- | --- | --- | --- |
|  | **AB** | **Ba** | **Ce** | **Co** | **La** | **Me** | **PHA** |  | **AB** | **Ba** | **Ce** | **Co** | **La** | **Me** | **PHA** |
| **AB** | NA |  |  |  |  |  |  |  | NA |  |  |  |  |  |  |
| **Ba** | 1.000 | NA |  |  |  |  |  |  | 1.000 | NA |  |  |  |  |  |
| **Ce** | 1.000 | 1.000 | NA |  |  |  |  |  | 0.922 | 0.855 | NA |  |  |  |  |
| **Co** | 1.000 | 1.000 | 1.000 | NA |  |  |  |  | 0.999 | 0.995 | 0.995 | NA |  |  |  |
| **La** | 1.000 | 1.000 | 1.000 | 1.000 | NA |  |  |  | 0.999 | 1.000 | 0.703 | 0.969 | NA |  |  |
| **Me** | 1.000 | 1.000 | 1.000 | 1.000 | 1.000 | NA |  |  | 0.961 | 0.920 | 1.000 | 0.999 | 0.803 | NA |  |
| **PHA** | 1.000 | 1.000 | 1.000 | 1.000 | 1.000 | 1.000 | NA |  | 0.160 | 0.126 | 0.691 | 0.340 | 0.068 | 0.659 | NA |
|  |  |  |  |  |  |  |  |  |  |  |  |  |  |  |  |
|  | ***DLB*** | | | | | | |  | ***AD+LB*** | | | | | | |
|  | **AB** | **Ba** | **Ce** | **Co** | **La** | **Me** | **PHA** |  | **AB** | **Ba** | **Ce** | **Co** | **La** | **Me** | **PHA** |
| **AB** | NA |  |  |  |  |  |  |  | NA |  |  |  |  |  |  |
| **Ba** | 1.000 | NA |  |  |  |  |  |  | 1.000 | NA |  |  |  |  |  |
| **Ce** | 0.432 | 0.453 | NA |  |  |  |  |  | **0.003** | **0.010** | NA |  |  |  |  |
| **Co** | 0.914 | 0.929 | 0.063 | NA |  |  |  |  | 0.990 | 1.000 | **0.030** | NA |  |  |  |
| **La** | 1.000 | 1.000 | 0.448 | 0.904 | NA |  |  |  | 0.885 | 0.979 | 0.104 | 0.999 | NA |  |  |
| **Me** | 0.583 | 0.604 | 1.000 | 0.099 | 0.598 | NA |  |  | **0.001** | **0.001** | 0.303 | **0.002** | **0.004** | NA |  |
| **PHA** | **1.000** | **1.000** | 0.687 | 0.777 | 1.000 | 0.826 | NA |  | 0.949 | 1.000 | **0.003** | 0.990 | 0.885 | **0.001** | NA |

C) *p-tau pathology between nuclei within groups*

|  | ***iLBD*** | | | | | | |  | ***PD*** | | | | | | |
| --- | --- | --- | --- | --- | --- | --- | --- | --- | --- | --- | --- | --- | --- | --- | --- |
|  | **AB** | **Ba** | **Ce** | **Co** | **La** | **Me** | **PHA** |  | **AB** | **Ba** | **Ce** | **Co** | **La** | **Me** | **PHA** |
| **AB** | NA |  |  |  |  |  |  |  | NA |  |  |  |  |  |  |
| **Ba** | 0.816 | NA |  |  |  |  |  |  | 0.183 | NA |  |  |  |  |  |
| **Ce** | 1.000 | 0.941 | NA |  |  |  |  |  | 1.000 | 0.137 | NA |  |  |  |  |
| **Co** | 0.811 | 0.083 | 0.740 | NA |  |  |  |  | 0.985 | 0.649 | 0.962 | NA |  |  |  |
| **La** | 0.991 | 0.272 | 0.972 | 0.993 | NA |  |  |  | 0.758 | **0.002** | 0.863 | 0.261 | NA |  |  |
| **Me** | 0.411 | **0.026** | 0.337 | 0.980 | 0.786 | NA |  |  | 0.072 | **0.00** | 0.117 | **0.008** | 0.755 | NA |  |
| **PHA** | 1.000 | 0.887 | 1.000 | 0.828 | 0.990 | 0.423 | NA |  | **0.000** | 0.107 | **0.000** | **0.001** | **0.000** | **0.000** | NA |
|  |  |  |  |  |  |  |  |  |  |  |  |  |  |  |  |
|  | ***DLB*** | | | | | | |  | ***AD+LB*** | | | | | | |
|  | **AB** | **Ba** | **Ce** | **Co** | **La** | **Me** | **PHA** |  | **AB** | **Ba** | **Ce** | **Co** | **La** | **Me** | **PHA** |
| **AB** | NA |  |  |  |  |  |  |  | NA |  |  |  |  |  |  |
| **Ba** | 0.463 | NA |  |  |  |  |  |  | 0.997 | NA |  |  |  |  |  |
| **Ce** | 1.000 | 0.300 | NA |  |  |  |  |  | 0.051 | **0.008** | NA |  |  |  |  |
| **Co** | 0.874 | **0.035** | 0.974 | NA |  |  |  |  | 0.608 | 0.240 | 0.873 | NA |  |  |  |
| **La** | 0.991 | 0.114 | 1.000 | 0.998 | NA |  |  |  | 0.923 | 0.605 | 0.515 | 0.997 | NA |  |  |
| **Me** | **0.031** | **0.000** | 0.093 | 0.513 | 0.187 | NA |  |  | **0.001** | **0.000** | 0.368 | **0.048** | **0.013** | NA |  |
| **PHA** | 0.171 | 0.996 | 0.086 | **0.006** | **0.027** | **0.000** | NA |  | 0.973 | 1.000 | **0.014** | 0.226 | 0.522 | **0.000** | NA |

D) *Granular cytoplasmic neuronal tau inclusion density between nuclei within groups*

|  | ***iLBD*** | | | | | | |  | ***PD*** | | | | | | |
| --- | --- | --- | --- | --- | --- | --- | --- | --- | --- | --- | --- | --- | --- | --- | --- |
|  | **AB** | **Ba** | **Ce** | **Co** | **La** | **Me** | **PHA** |  | **AB** | **Ba** | **Ce** | **Co** | **La** | **Me** | **PHA** |
| **AB** | NA |  |  |  |  |  |  |  | NA |  |  |  |  |  |  |
| **Ba** | 0.442 | NA |  |  |  |  |  |  | 0.726 | NA |  |  |  |  |  |
| **Ce** | 0.994 | 0.790 | NA |  |  |  |  |  | 0.983 | 0.304 | NA |  |  |  |  |
| **Co** | 0.970 | 0.913 | 1.000 | NA |  |  |  |  | 1.000 | 0.860 | 0.937 | NA |  |  |  |
| **La** | 0.994 | 0.698 | 1.000 | 1.000 | NA |  |  |  | 0.137 | 0.902 | **0.038** | 0.221 | NA |  |  |
| **Me** | 1.000 | 1.000 | 1.000 | 1.000 | 1.000 | NA |  |  | 0.994 | 0.490 | 1.000 | 0.972 | 0.117 | NA |  |
| **PHA** | 0.509 | 1.000 | 0.865 | 0.927 | 0.811 | 1.000 | NA |  | 0.072 | 0.723 | **0.020** | 0.118 | 1.000 | 0.063 | NA |
|  |  |  |  |  |  |  |  |  |  |  |  |  |  |  |  |
|  | ***DLB*** | | | | | | |  | ***AD+LB*** | | | | | | |
|  | **AB** | **Ba** | **Ce** | **Co** | **La** | **Me** | **PHA** |  | **AB** | **Ba** | **Ce** | **Co** | **La** | **Me** | **PHA** |
| **AB** | NA |  |  |  |  |  |  |  | NA |  |  |  |  |  |  |
| **Ba** | 0.798 | NA |  |  |  |  |  |  | 0.393 | NA |  |  |  |  |  |
|  | 1.000 | 0.678 | NA |  |  |  |  |  | 0.697 | **0.007** | NA |  |  |  |  |
| **Co** | 1.000 | 0.734 | 1.000 | NA |  |  |  |  | 0.691 | 0.999 | 1.000 | NA |  |  |  |
| **La** | 0.971 | 0.998 | 0.922 | 0.945 | NA |  |  |  | 0.917 | 0.972 | 0.922 | 0.945 | NA |  |  |
| **Me** | 0.478 | 0.119 | 0.569 | 0.542 | 0.205 | NA |  |  | 0.227 | **0.006** | 0.569 | 0.542 | 0.205 | NA |  |
| **PHA** | 0.910 | 1.000 | 0.820 | 0.860 | 1.000 | 0.157 | NA |  | 1.000 | 0.781 | 0.820 | 0.860 | 1.000 | 0.157 | NA |

Post-hoc pairwise comparisons for Aβ pathology (total %area of 4G8 immunoreactivity) (A), diffuse plaque density (in number per 0.64 mm^2^) (B), p-tau pathology (total % area of AT8 immunoreactivity) (C) and granular cytoplasmic neuronal tau inclusion density (in number per 0.25 mm^2^) (D) between amygdalar nuclei within groups were computed using estimated marginal means with Tukey’s method for multiple comparisons. Displayed are the p-values that correspond to these adjusted pairwise contrasts. Statistically significant (p<0.05) comparisons are indicated in bold. *AB: accessory basal nucleus; AD+LB: Alzheimer’s disease with Lewy bodies; Ba: basal nucleus; Ce: central nucleus; Co: cortical nucleus; DLB: dementia with Lewy bodies; iLBD: incidental Lewy body disease; La: lateral nucleus; Me: medial nucleus; NA: not applicable; PD: Parkinson’s disease; PHA: parahippocampal-amygdaloid transition area.*

1.	Postuma, R.B., et al., *MDS clinical diagnostic criteria for Parkinson's disease.* Mov Disord, 2015. **30**(12): p. 1591–601.

2.	Morris, J.C., *The Clinical Dementia Rating (CDR): current version and scoring rules.* Neurology, 1993. **43**(11): p. 2412–4.

3.	McKeith, I.G., *Clinical Lewy body syndromes.* Ann N Y Acad Sci, 2000. **920**: p. 1–8.

4.	Moors, T.E. and D. Milovanovic, *Defining a Lewy Body: Running Up the Hill of Shifting Definitions and Evolving Concepts.* J Parkinsons Dis, 2024. **14**(1): p. 17–33.

5.	Otero-Jimenez, M., et al., *Neuropathological stages of neuronal, astrocytic and oligodendrocytic alpha-synuclein pathology in Parkinson's disease.* Acta Neuropathol Commun, 2025. **13**(1): p. 25.

6.	Smith, C., et al., *Greenfield's Neuropathology 10e Set*. 2024: CRC Press.

7.	Tschanz, J.T., *Amyloid Plaques*, in *Encyclopedia of Clinical Neuropsychology*, J.S. Kreutzer, J. DeLuca, and B. Caplan, Editors. 2011, Springer. p. 155.

8.	Liu, F., et al., *Focal-type, but not Diffuse-type, Amyloid Beta Plaques are Correlated with Alzheimer's Neuropathology, Cognitive Dysfunction, and Neuroinflammation in the Human Hippocampus.* Neurosci Bull, 2022. **38**(10): p. 1125–1138.

9.	Boon, B.D.C., et al., *The coarse-grained plaque: a divergent Abeta plaque-type in early-onset Alzheimer's disease.* Acta Neuropathol, 2020. **140**(6): p. 811–830.

10.	Tome, S.O., et al., *Distinct molecular patterns of TDP-43 pathology in Alzheimer's disease: relationship with clinical phenotypes.* Acta Neuropathol Commun, 2020. **8**(1): p. 61.

11.	Nelson, P.T., et al., *LATE-NC staging in routine neuropathologic diagnosis: an update.* Acta Neuropathol, 2023. **145**(2): p. 159–173.

12.	Hansra, G.K., et al., *The neuritic plaque in Alzheimer's disease: perivascular degeneration of neuronal processes.* Neurobiol Aging, 2019. **82**: p. 88–101.
